# The basic keratin 10-binding domain of the virulence-associated pneumococcal serine-rich protein PsrP adopts a novel MSCRAMM fold

**DOI:** 10.1098/rsob.130090

**Published:** 2014-01-15

**Authors:** Tim Schulte, Jonas Löfling, Cecilia Mikaelsson, Alexey Kikhney, Karina Hentrich, Aurora Diamante, Christine Ebel, Staffan Normark, Dmitri Svergun, Birgitta Henriques-Normark, Adnane Achour

**Affiliations:** 1 Science for Life Laboratory, Center for Infectious Medicine (CIM), Department of Medicine, Karolinska University Hospital Huddinge, Karolinska Institutet Science Park, Tomtebodavägen 23A Solna, Stockholm 17165, Sweden; 2 Department of Microbiology, Tumor and Cell Biology (MTC), Karolinska Institutet, Stockholm, Sweden; 3 Department of Laboratory Medicine, Division of Clinical Microbiology, Karolinska University Hospital Solna, Stockholm, Sweden; 4 European Molecular Biology Laboratory (EMBL), Hamburg Outstation, Notkestrasse 85, Hamburg 22603, Germany; 5 Institut de Biologie Structurale, CEA-CNRS-Université, Grenoble, France

**Keywords:** bacterial virulence factor, PsrP, *Streptococcus pneumoniae*, adhesion, MSCRAMM, keratin-10

## Abstract

*Streptococcus pneumoniae* is a major human pathogen, and a leading cause of disease and death worldwide. Pneumococcal invasive disease is triggered by initial asymptomatic colonization of the human upper respiratory tract. The pneumococcal serine-rich repeat protein (PsrP) is a lung-specific virulence factor whose functional binding region (BR) binds to keratin-10 (KRT10) and promotes pneumococcal biofilm formation through self-oligomerization. We present the crystal structure of the KRT10-binding domain of PsrP (BR_187–385_) determined to 2.0 Å resolution. BR_187–385_ adopts a novel variant of the DEv-IgG fold, typical for microbial surface components recognizing adhesive matrix molecules adhesins, despite very low sequence identity. An extended β-sheet on one side of the compressed, two-sided barrel presents a basic groove that possibly binds to the acidic helical rod domain of KRT10. Our study also demonstrates the importance of the other side of the barrel, formed by extensive well-ordered loops and stabilized by short β-strands, for interaction with KRT10.

## 2. Introduction

*Streptococcus pneumoniae* (pneumococcus) is a human-adapted, Gram-positive commensal bacterium that colonizes the upper respiratory tract in about 10% of healthy adults and up to 60% of children. Although normally not causing any symptoms, pneumococcus is a major human pathogen, and a leading cause of disease and death worldwide [1]. Streptococcal antigenicity is determined to a large extent by the structure and contents of the outermost layer of the cell, including a variety of proteins with differing functions localized within the polysaccharide capsule [1,2]. Surface-associated adhesins play a pivotal role for pneumococcal colonization of the nasopharynx and for the development of infectious pneumococcal disease through interactions with specific cellular surface structures in the host [3].

**Figure 1.**
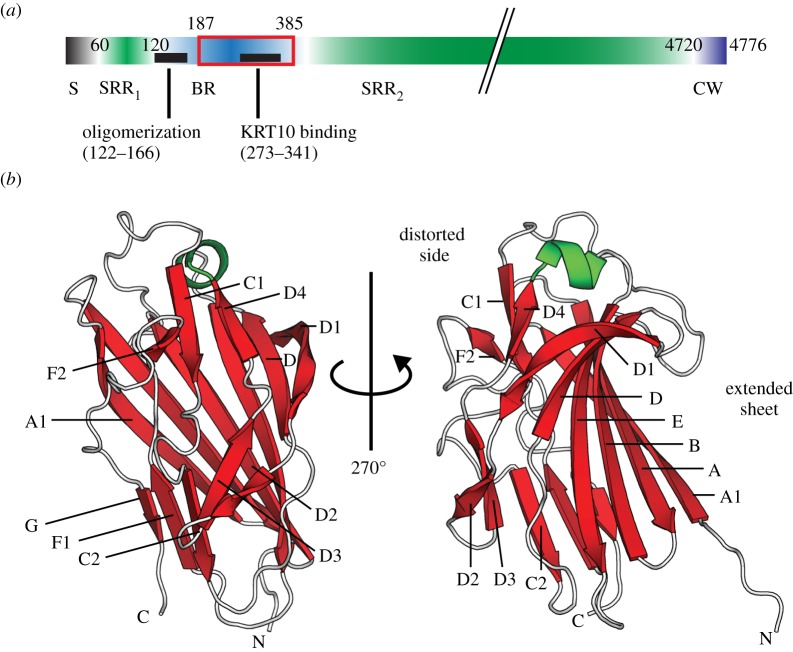
Crystal structure of the KRT10-binding region of PsrP (BR_187–385_). (*a*) PsrP is organized into five domains, comprising the N-terminal signal sequence (S) for export of PsrP to the extracellular surface, two serine-rich repeat regions (SRR_1_ and SRR_2_), the binding region (BR) domain and the cell wall (CW) domain. The basic BR domain harbours two distinct subregions for KRT10-binding (black bar, residues 273–341) and self-oligomerization (black bar, residues 122–166), respectively. BR_187–385_ contains only the KRT10-binding subregion. (*b*) Two orientations of the BR_187–385_ crystal structure are presented as ribbon diagrams with β-strands, α-helices and loops in red, green and white, respectively. While one side of the compressed barrel of BR_187–385_ is created by an extended and twisted antiparallel β-sheet, the other side is formed by well-ordered loops and two sets of β-sheet belts.

The pneumococcal serine-rich repeat protein (PsrP) is an important lung-specific virulence factor that is present in 60% of strains capable of causing pneumonia in children [2]. The C-terminal cell wall anchoring domain of PsrP contains an LPxTG motif that is covalently anchored to the peptidoglycan by Sortases [4]. A characteristic feature of the serine-rich repeat protein (SRRP) family is the presence of a long, highly repetitive and glycosylated C-terminal serine-rich repeat (SRR) region (figure 1*a*) that can vary between 400 and 4000 residues [5]. The size of the possibly super-helical SRR region might correlate with the capsule thickness of each species, extending the highly basic functional binding region (BR) domain of each SRRP out of the capsule [5–8]. The sequence of the BR domain, which includes the N-terminal SRR_1_ and the longer C-terminal SRR_2_ regions, is extremely variable among all known SRRPs, which could account for the broad range of targets bound by this adhesin family [5,8]. Pneumococcal PsrP promotes both biofilm formation through self-oligomerization and adherence to keratin 10 (KRT10)-expressing lung epithelial cells. These disparate functions are facilitated by two distinct regions within the surface-exposed BR domain [7,9].

Keratins (KRTs) are intermediary filament (IF) proteins that are mainly regarded as intracellular constituents of the cytoskeleton [10]. More than 50 distinct human KRT genes are expressed in a highly cell-type- and cell-differentiation-state-dependent manner [11]. All KRTs exhibit a tripartite structure characterized by a long α-helical rod domain flanked by an amino- and a carboxy-terminal non-α-helical end domain. The secondary structure of the rod domain, which is highly conserved among IF proteins, is divided into four heptad-repeat-containing helical segments called 1A, 1B, 2A and 2B, which are interrupted by three short linker sequences L1, L12 and L2 [10]. The heptad-repeat-containing segments form the structural basis for the heteromeric assembly of KRT filaments [12]. For example, the acidic type-I KRT-10 and the basic type-II KRT-1 form an obligate heterodimer that is the main building block in filament assembly.

It has been previously demonstrated that KRTs are also readily available on the surface of epithelial cells, acting as potential surface-accessible docking sites for microbial adhesins. While the *Staphylococcus aureus*-derived adhesin clumping factor B (ClfB) interacts with KRT10 and possibly KRT8 on the surface of desquamated stratified squamous epithelial cells isolated from human nares [13,14], the *Streptococcus agalactiae*-derived SRR-1 protein interacts with KRT4 on the surface of human laryngeal carcinoma-derived Hep2-cells [15].

In the structurally and mechanistically well-described ‘dock, lock and latch’ binding mode of the microbial surface components recognizing adhesive matrix molecules (MSCRAMMs) ClfB to KRT10, a peptide derived from the tail of KRT10 ‘docks’ into a binding trench localized between the two homologous subdomains N2 and N3 of ClfB, and undergoes a disorder-to-order transition by complementing a β-sheet within N3. The C-terminal extension of N3 is thereafter redirected in order to ‘lock’ the KRT10 peptide in place and to form a ‘latch’ through β-sheet complementation with N2 [16,17].

Both the N2 and N3 domains of ClfB display the DE-variant of the IgG fold (DEv-IgG) that has been described for the A-region of the *S. aureus*-derived CNA protein [18,19]. Interestingly, CnaA subdomains with similar topology have been identified in the two other available crystal structures of SRRPs, Fap1 and GspB, derived from *Streptococcus parasanguinis* and *Streptococcus gordonii*, respectively [6,8]. The presence of a CnaA-subdomain has also been predicted for the *Streptococcus agalactiae*-derived KRT4-binding SRRP SRR-1 [8]. However, the topology for the BR domain of PsrP could not be predicted owing to missing sequence homology. In this study, the crystal structure of the KRT10-binding region of PsrP (BR_187–385_) was determined, revealing a novel fold distantly related to CnaA subdomains. While one face of the compressed, two-sided barrel of BR_187–385_ is created by an extended β-sheet that presents a highly basic binding groove, extensive well-ordered loop regions distort the other face of the barrel, forming a paperclip-like substructure. *In vitro* alanine substitution of residues localized within this paperclip structure efficiently disrupted BR_187–385_/KRT10 complex formation.

## 3. Results and discussion

### 3.1. The crystal structure of monomeric BR_187–385_ presents a compressed β-barrel fold with two remarkably different faces

BR_187–385_ crystallized in two crystal forms of the space groups P4_3_2_1_2 and P4_1_22, with differing unit cell parameters (table 1). Single anomalous dispersion (SAD) data, collected from a seleno-methionine derivative of the P4_3_2_1_2 crystal form that diffracted to 2.25 Å, was used to solve the phase problem. Three BR_187–385_ polypeptide chains were placed in the asymmetric unit, and refined to *R-* and *R*_free_-values of 18.6% and 21.5%, respectively. The native P4_1_22 dataset that diffracted to 2.0 Å was solved using BR_187–385_ from P4_3_2_1_2 as a template for molecular replacement (MR). A single BR_187–385_ molecule was found in the P4_1_22 asymmetric unit, and a model comprising residues L203–S378 was refined to *R-* and *R*_free_-values of 17.7% and 20.1%, respectively. The structural deviation between the P4_1_22-BR_187–385_ monomer and each of the three P4_3_2_1_2-BR_187–385_ molecules was minimal, with root mean square deviations (r.m.s.d.) of 0.6 Å and less than 0.4 Å, following superposition of the P4_1_22 monomer on the P4_3_2_1_2 chains A and B/C, respectively.

**Table 1.** X-ray data collection and refinement statistics.

	SeMet	native
data collection		
space group	P4_3_2_1_2	P4_1_22
unit cell parameters (*a*, *b*, *c* in Å; *α*, *β*, *γ* in degree)	*a* = 105.6; *b* = 105.6; *c* = 120. 5; α = β = γ = 90	*a* = 74.5; *b* = 74.5; *c* = 121.2; *α* = *β* = *γ* = 90
X-ray source	ESRF ID-29	ESRF ID-29
detector	Pilatus 6M	Pilatus 6M
temperature (K)	100	100
resolution limits (Å)	48.35–2.25 (2.31–2.25)	48.32–2.00 (2.05–2.00)
wavelength (Å)	0.979	0.977
no. of observations	283775 (20035)	296169 (21744)
no. of unique reflections	61680 (4563)	44053 (3252)
redundancy	4.6 (4.4)	6.7 (6.7)
completeness (%)	100 (100)	100 (100)
*I*/*σ*	14.83 (3.18)	18.18 (2.96)
*R*_sym_ (%)	6.5 (48.7)	6.3 (69.5)
*R*_meas_ (%)	7.3 (55.5)	6.9 (75.4)
*R*_mrgd-F_ (%)	10.6 (52.8)	7.4 (53.5)
phasing		
software	SHELX [20]	
resolution limit	20–2.25	
no. of sites found	12	
CC all/weak	60.46/40.35	
FOM after SHELXE	0.707	
FOM after DM	0.904	
structure refinement		
PDB entry	3ZGI	3ZGH
molecules in ASU	3	1
mask estimated solvent (%)	56	68
*R*_work_ (%)	18.6	17.7
*R*_free_ (%)	21.5	20.1
no. of residues		
protein	522	177
water	146	114
small molecule	6	2
other	1	4
no. of atoms	4171	1494
mean isotropic B-value (Å²)	51.4	42.5
Wilson B-factor (Å²)	36.1	35.3
r.m.s.d. from ideal bond lengths (Å)	0.01	0.012
r.m.s.d. from ideal bond angle (°)	1.47	1.417
molprobity		
Ramachandran		
outliers (%)	1.4	0.6
favoured (%)	96	95
all atom clash score (percentile)	6.44 (98th)	8.44 (89th)
molprobity score (percentile)	1.65 (97th)	1.87 (82nd)

The overall three-dimensional structure of BR_187–385_, composed of 43% β-strands, 2% α-helices, 17% turns and 38% loop regions, can be described as a compressed barrel with two remarkably different faces (figure 1*b*). While one side of the barrel forms an extended and twisted antiparallel β-sheet that comprises the six strands A1, A, B, E, D and D1, the other side mainly consists of well-ordered loops, each stabilized by two sets of β-sheet belts, comprising strands D2, D3, C2, F1, G and D4, C1, F2, respectively. Furthermore, the highly ordered loops are also stabilized by several β-turns and hairpin motifs (data not shown).

**Figure 2.**
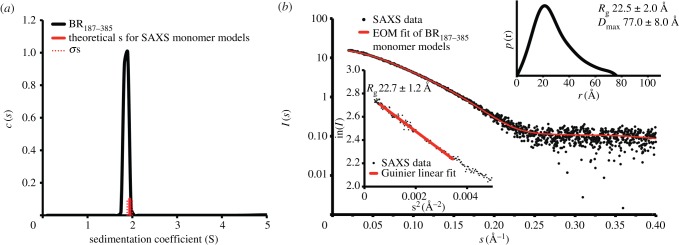
BR_187–385_ is a monomer in solution. (*a*) The continuous distribution of sedimentation coefficients reveals a single population for the BR_187–385_ monomer with a sedimentation coefficient of 1.85 S. The averaged theoretical sedimentation coefficients calculated for the SAXS-derived BR_187–385_ monomer models are in agreement with the experimentally determined value. The averaged value and the standard deviation are in red and dotted red, respectively. (*b*) The theoretical scattering curve obtained for an ensemble of 18 BR_187–385_ monomer models (see also electronic supplementary material, figure S1) fits the SAXS profile of BR_187–385_ with a *χ*^2^-value of 0.58. The radius of gyration *R*_g_ obtained from the Guinier approximation was 22.7 ± 1.2 Å (mean ± s.d.). The distance distribution function *p*(*r*) gave *R*_g_ and *D*_max_ values of 22.5 ± 2.0 Å and 77.0 ± 8.0 Å, respectively.

Crystal packing analysis revealed that two symmetry-related molecules in the P4_1_22 crystal formed an intermolecular β-sheet resulting in an interface area of 585 Å^2^. The same interface was also observed for chains B and C in the P4_3_2_1_2 crystal form. However, a single population with a sedimentation coefficient of 1.85 S corresponding to a monomer with a hydrodynamic radius of 25 Å was clearly assessed using analytical ultracentrifugation (AUC; figure 2*a*). A similar hydrodynamic radius value was also derived from the retention volume of the BR_187–385_ monomer using size exclusion chromatography (SEC; data not shown). Finally, small angle X-ray scattering (SAXS) analysis of BR_187–385_ revealed a monomer in solution with a molecular weight estimated from the forward scattering *I*(0) to 18 ± 2 kDa and from the Porod volume to 23 ± 2 kDa (expected at 22 kDa; figure 2*b*; electronic supplementary material, table S1). While the radii of gyration *R*_g_ obtained from the Guinier approximation and from the distance distribution function *p*(*r*) were 22.7 ± 1.2 Å and 22.5 ± 2.0 Å, respectively, the *D*_max_ value was 77.0 ± 8.0 Å. It should be noted that the extended *p*(*r*) function suggested a partially unfolded protein. Furthermore, fitting of the experimental data using the ensemble optimization method (EOM) also indicated the formation of a globular envelope with N- and C-terminal extensions (see electronic supplementary material, figure S1). The ensemble of 18 monomer models yielded a theoretical average sedimentation coefficient of 1.94 ± 0.06 S, in good agreement with the AUC analysis (figure 2*a*).

In conclusion, the crystal structure of the BR_187–385_ monomer takes a compressed barrel fold with one face formed by an extended and twisted β-sheet, and the other face mainly consisting of well-ordered loops. We believe, at the present stage, that the formed pseudo-complexes are probably owing to crystal packing.

### 3.2. The KRT10-binding region domain of PsrP adopts a novel MSCRAMM fold-variant

**Figure 3.**
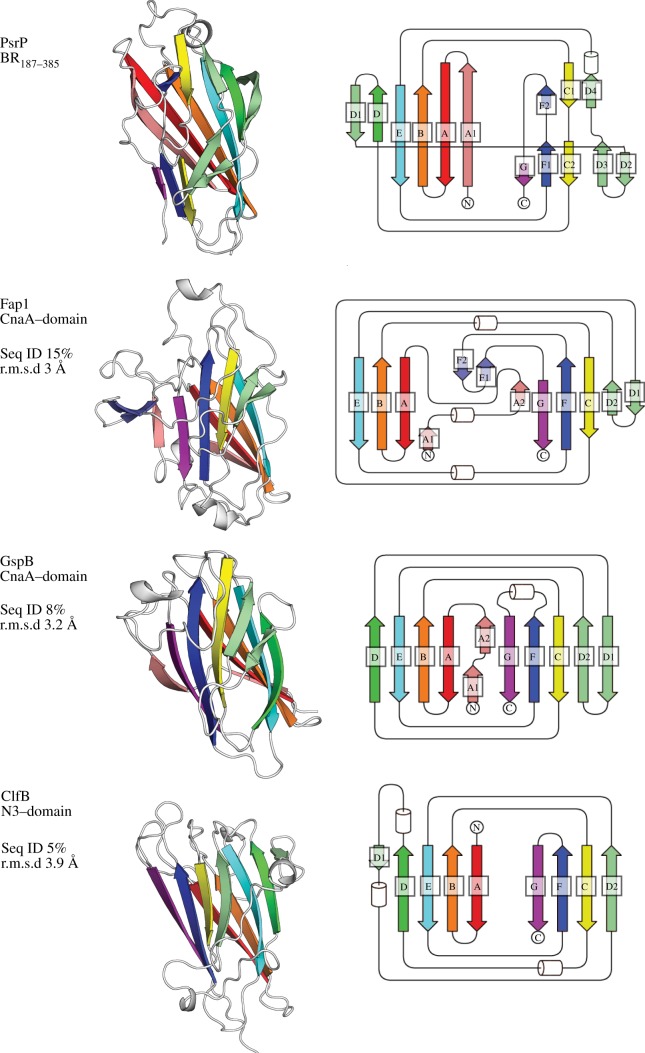
BR_187–385_ adopts a fold that is distantly related to the MSCRAMM-typical DEv-IgG fold. Both CnaA-subdomains of Fap1 (PDB: 2X12) and GspB (3QD1), as well as the N3 subdomain of ClfB (3AU0), superimpose to BR_187–385_ with r.m.s.d. values of 3.0, 3.2 and 3.9 Å, despite sequence identities of only 15%, 8% and 5% (see electronic supplementary material, figure S2 and supplemental movies), respectively. The fold of BR_187–385_ is distantly related to the canonical DEv-IgG fold displayed by the N3 domain of ClfB. Topology diagrams of the DEv-IgG fold variants are designated and colour-coded according to [8,19,21].

A search for structural homologues revealed that BR_187–385_ adopts an MSCRAMM-related DEv-IgG fold (figure 3). The typical DEv-IgG fold topology can be described as a compressed barrel composed of two opposing β-sheets that are formed by β-strands ABED (sheet I) and CFG (sheet II) [19]. The insertion of two extra strands between strands D and E distinguishes the DEv-IgG variant from the IgG-constant domain [22]. BR_187–385_ takes a novel DEv-IgG fold variant with one side of the barrel distorted by loops and β-turns, as well as extensive insertions of shorter strands and loops between strands D and E (figure 3).

The first nine structural homologues, identified using Dali [23], belonged to the MSCRAMM or SRRP family (see electronic supplementary material, table S2). Although they shared only 5–17% sequence identity with BR_187–385_, they all superimposed to BR_187–385_ with r.m.s.d. values stretching from 3.0 to 4.7 Å. In particular, both crystal structures of the CnaA-subdomains of the SRRPs Fap1 and GspB superimposed to BR_187–385_ with r.m.s.d. values of 3.0 and 3.2 Å, despite sequence identities of only 15% and 8%, respectively (see electronic supplementary material, table S2 and figure S2). Compared with GspB, the CnaA subdomains of Fap1 and BR_187–385_ are more distantly related to the canonical DEv-IgG fold displayed by ClfB (figure 3). While both BR_187–385_ and Fap1 bind using their CnaA-like subdomains to their cognate ligands KRT10 and saliva-coated hydroxylapatite, respectively, GspB binds the carbohydrate ligand sialyl T-antigen via its Siglec subdomain [6,8]. The *S. aureus*-derived ClfB was also chosen for comparison with BR_187–385_ (see electronic supplementary material, table S2 and figure S2) because it binds to a KRT10-derived linear peptide motif (LPM) via the ‘dock, lock and latch’ mechanism [16,17]. Here again, despite a sequence identity of only 5%, the N3 subdomain of ClfB superimposed to BR_187–385_ with an r.m.s.d. value of 3.9 Å (figure 3; electronic supplementary material, figure S2). However, superimposition of ClfB and BR_187–385_ also revealed that BR_187–385_ can probably not bind to KRT10 via the same ‘dock, lock and latch’ binding mode, because KRT10 is bound on different sites of the two proteins (see electronic supplementary material, figure S3).

### 3.3. The KRT10-binding region of BR_187–385_ resembles a paperclip

**Figure 4.**
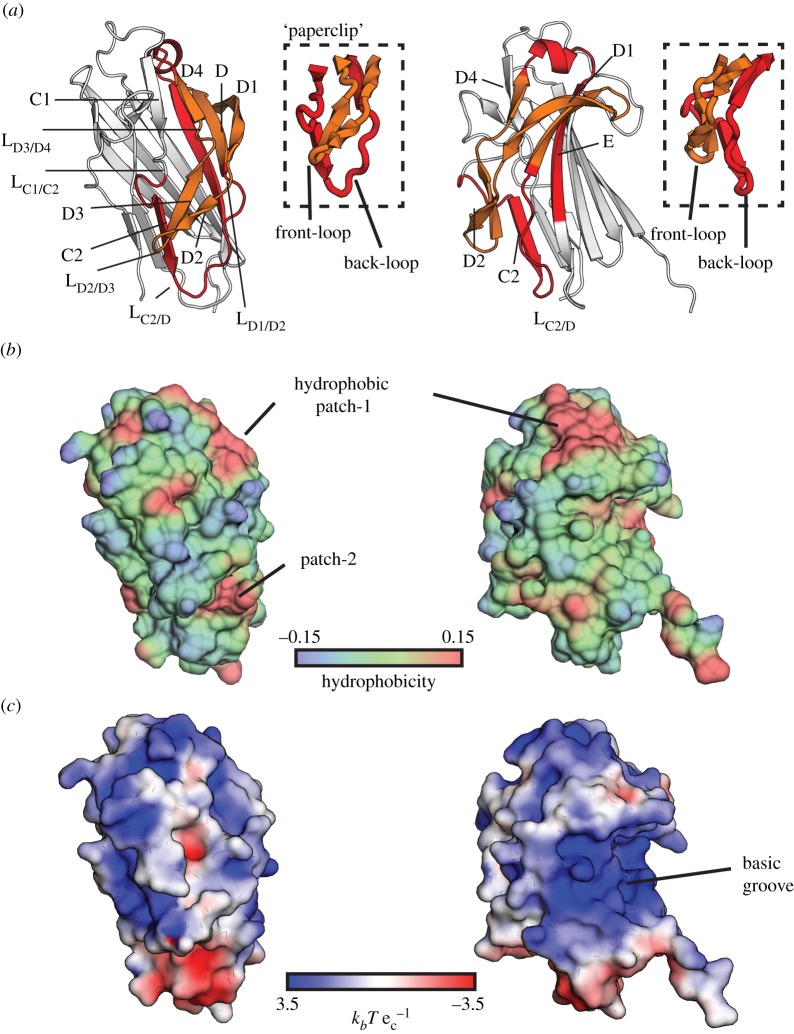
BR_187–385_ comprises a paperclip-like region and a basic binding groove for interaction with KRT10. (*a*) The putative paperclip region provides an explanation for previous experimental observations [9]. The back- and front-loops of the clip, formed by residues 268–295 and 305–324, are coloured red and orange, respectively. Boxes highlight this specific region. Binding of KRT10 most probably requires conformational re-arrangements of the three loops L_C1/C2_, L_D1/D2_ and L_D3/D4_ (see electronic supplementary material, figure S4). (*b*) Hydrophobic patches on BR_187–385_ are displayed on a surface hydrophobicity distribution plot. Regions coloured red and blue are hydrophobic (positive values) and hydrophilic (negative values), respectively. Two hydrophobic patches are localized within the KRT10-binding region. While hydrophobic patch-1 is localized proximally to strand D4, hydrophobic patch-2 is localized underneath the tip of the front-loop of the clip. Hydrophobicity is plotted on a relative scale. (*c*) Analysis of the surface electrostatic potential of BR_187–385_ reveals a highly positively charged binding groove formed by the extended antiparallel β-sheet I that could accommodate the acidic helical rod domain of the KRT10/KRT1 heterodimer (see electronic supplementary material, figure S5). The electrostatic potential is plotted in 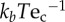 with the Boltzmann's constant *k_b_*, the charge of an electron e_c_ at a temperature of 298 K.

The KRT10-binding region of PsrP that comprises residues 273–341 corresponds to a region involving most of strand E, as well as strands C2, D, D1, D2, D3 and D4, all connected by the loops L_C2/D_, L_C1/C2_, L_D1/D2_, L_D2/D3_ and L_D3/D4_ (figure 4*a*). A substructure within this region takes a paperclip form with back- and front-loops formed by residues 268–295 and 305–324, respectively, which provides a possible explanation to previous experimental observations (figure 4*a*) [9]. Indeed, while pre-incubation of KRT10^+^-A549 cells with a BR-construct comprising residues 273–341 (front- and back-loops) blocked binding of pneumococcal TIGR4, pre-incubation with a shorter BR construct stretching from residues 291 to 325 (front-loop only) resulted in binding to KRT10, but did not block binding of TIGR4 bacteria to A549 cells.

We hypothesized that binding of KRT10 may require conformational re-arrangements of the three loops L_C1/C2_, L_D1/D2_ and L_D3/D4_ (figure 4*a*). Analysis of the distribution of B-factor values revealed a high mobility of L_C1/C2_ and L_D3/D4_, as well as of L_D2/D3_ localized at the tip of the front-loop, compared with the rest of the structure (see electronic supplementary material, figure S4*a*). Rigidity analysis of BR_187–385_ confirmed that L_C1/C2_ did not belong to the single large rigid cluster formed by almost the entire BR_187–385_ structure (see electronic supplementary material, figure S4*b*). Our analysis indicated that L_D3/D4_ could be uncoupled by breakage of a single hydrogen bond interaction between the backbone oxygen of the asparagine residue N321 and the hydroxyl group of the serine residue S308. Furthermore, removal of three and four hydrogen bond interactions between the front- and back-loop regions would uncouple L_D1/D2_ and the entire front-loop region from the rigid cluster, respectively.

Hydrophobic interactions represent an essential mechanism for binding to intrinsically disordered protein regions [24] such as KRT10-associated glycine loops. Two distinct solvent-accessible hydrophobic pockets, localized proximally to strand D4 and underneath the front-loop, could possibly act as initial anchor points for interaction (figure 4*b*). Furthermore, inspection of the electrostatic surface of BR_187–385_ revealed the presence of a highly basic groove with a solvent-accessible surface area of 180 Å^2^ (figure 4*c*) that could easily accommodate the elongated and negatively charged helical rod domain of KRT10 (see electronic supplementary material, figure S5). The complementary charges of the basic BR_187–385_ and the acidic rod domains of KRT10/KRT1 (with theoretical isoelectric points of 4.6 and 4.8, respectively) could play an important role in initial complex formation, because electrostatic interactions are dominant long-range forces for protein associations [25]. Interestingly, the functional binding domain of the *S. agalactiae*-derived SRR-1 probably contains a CnaA-like domain, as predicted by sequence homology [8]. This CnaA subdomain with a theoretical isoelectric point of 4.7 binds to the carboxy-terminal domain of keratin-4 (KRT4) that belongs to basic type-II IFs [15]. This suggests that the surface of the two hitherto known keratin-binding SRRPs, PsrP and SRR-1, could be charge-optimized for efficient binding to oppositely charged IF protein ligands.

In conclusion, our structural analysis suggests that the KRT10-minimal binding region of BR_187–385_ resembles a paperclip that could allow for binding to KRT10 following conformational rearrangements of the clip-associated loops. Furthermore, the extended β-sheet on one side of the compressed barrel may provide a basic binding groove that could accommodate parts of the highly negatively charged helical rod domains of the KRT10/KRT1 heterodimer.

### 3.4. Binding of BR_187–385_ to keratin-10 is disrupted by alanine substitution of several residues within the paperclip region

**Figure 5.**
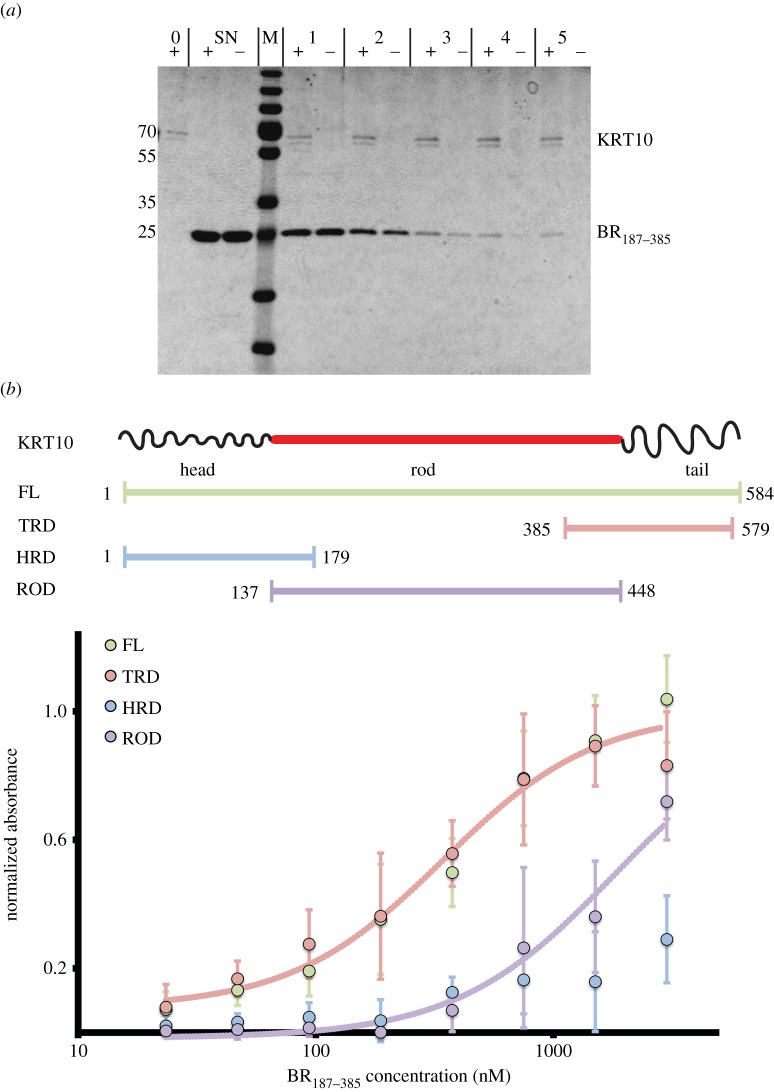
BR_187–385_ binds to the tail-rod-2B end of KRT10. (*a*) BR_187–385_ bound to KRT10-FL immobilized on Ni-NTA magnetic agarose beads (+), and a small fraction remained associated after washing, while BR_187–385_ incubated with empty beads (−) was completely removed following the fourth wash step. Beads were analysed using SDS–PAGE before incubation with BR_187–385_ (0), after 10 min wash using magnetic PBS buffer (1), 1 min wash using magnetic washing buffer (2) and three times of a 10 min wash using magnetic washing buffer (3–5). The supernatant of BR_187–385_ after incubation with the beads (SN) confirms that equal amounts of protein were used for the assay. (*b*) A scheme of the tripartite structure of the intermediate filament protein keratin-10 (KRT10) shows the glycine loops localized at both end domains that are separated by a α-helical rod domain. The KRT10-FL, KRT10-HRD, KRT10-TRD and KRT10-ROD constructs comprise the stretches of residues 1–584, 1–179, 385–579 and 137–448, respectively. While BR_187–385_ bound to both KRT10-FL and KRT10-TRD with a common EC_50_-value of 345 nM (256–460 nM at 95% CI, *R*^2^ of 0.82), binding to the KRT10-rod domain (KRT10-ROD) was significantly reduced with an estimated EC_50_-value of 1.8 μM (1.4–2.3 μM at 95% CI, *R*^2^ of 0.74). Binding to the KRT10 head-region-domain was at a much lower level (KRT10-HRD). Values are given as mean with 95% CI. EC_50_ values were derived using a single four-parameter logistic nonlinear regression model. The mean normalized immobilization levels for KRT10-FL, KRT10-HRD, KRT10-TRD and KRT10-ROD as determined by HRP-coupled anti-His antibodies were 1.0 ± 0.2 (mean ± s.d.), 1.1 ± 0.4, 0.7 ± 0.2 and 1.1 ± 0.2, respectively.

The interaction between BR_187–385_ and KRT10 was confirmed in a pull-down experiment in which Strep-Tag-II BR_187–385_ (STII-BR_187–385_) bound to Ni–NTA bead-immobilized full-length KRT10 (figure 5*a*). For ELISA assays, three shorter KRT10 constructs comprising the head and tail end regions (including the associated helical segments 1A and 2B from the rod domain, as well as the entire rod domain of KRT10) were produced. While STII-BR_187–385_ bound with similar capacity to both the KRT10-full-length (KRT10-FL) and the KRT10-tail-rod-2B (KRT10-TRD) domains with a shared EC_50_ value of 345 nM, binding to the KRT10-rod domain (KRT10-ROD) was significantly reduced, with an estimated EC_50_ value of 1.8 μM. Finally, binding of STII- BR_187–385_ to the KRT10 head-rod-1A domain (KRT10-HRD) was at a very low level (figure 5*b*).

Both head and tail domains are composed of glycine-rich loops, anchored to stacked arrays of aromatic and/or large apolar residues [10,26–28]. It has been previously suggested that, in contrast to the head domain, the tail of KRT10 is composed of fewer but larger (and therefore potentially more flexible) loop regions [27], which could play a key role in interactions with other proteins. For example, ClfB complements a KRT10 tail-derived LPM into a β-sheet, enforcing disorder-to-order transition of the peptide [16,17]. The peptide-binding site prediction server PepSite [29] identified two clip-associated binding sites in BR_187–385_ where several KRT10-derived LPMs could fit (see electronic supplementary material, figure S6). While binding site-1 was located within the first surface-accessible hydrophobic patch and localized proximally to strand D4, binding site-2 was localized underneath the front-loop region of the clip.

**Figure 6.**
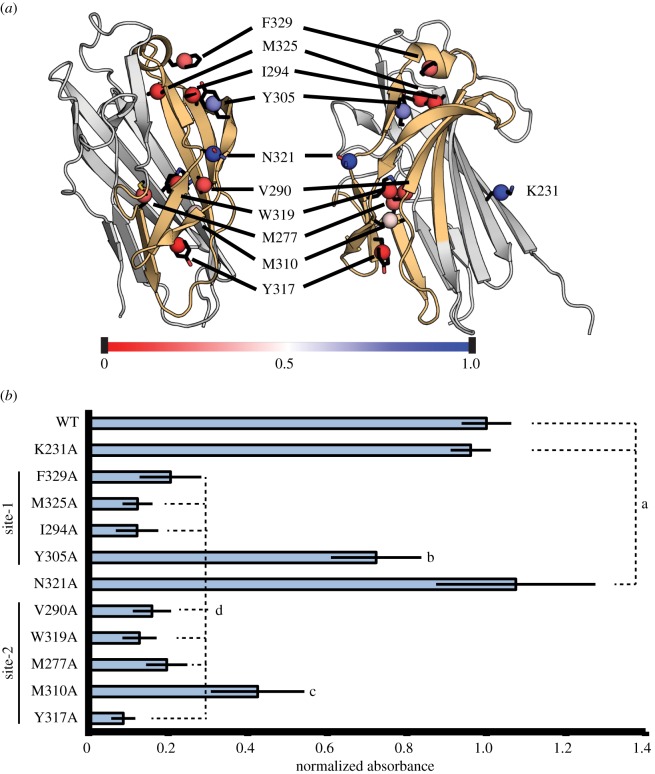
KRT10-TRD is bound in two contiguous paperclip-associated binding sites within BR_187–385_. (*a*) The location of each substituted residue is indicated. The centre of mass of each residue is displayed as a sphere and coloured according to its importance for binding to KRT10-TRD (red and blue for low and high binding intensities, respectively). The introduced substitutions did not significantly alter the overall secondary structure of the mutated BR_187–385_ proteins (see electronic supplementary material, figure S7). (*b*) Normalized intensity values were determined for binding of WT BR_187–385_ and each mutated variant to KRT10-TRD coated in wells of ELISA plates. Values are given as mean with standard deviation. WT and mutated BR_187–385_ variants were classified into groups a–d using the Tukey test at a significance level of *p* < 0.05.

Ten residues located within or in the near vicinity of the two predicted paperclip-associated binding sites of BR_187–385_ were substituted to alanine (figure 6*a*), and all mutated proteins were tested for binding to KRT10-TRD using ELISA. Residue K231, which is not located within the predicted binding sites, was selected as a negative control. The interaction levels were reduced by at least 80% compared with WT-BR_187–385_ and control K231A-BR_187–385_ for seven of 10 mutations, including residues F329, M325 and I294 localized in site-1, as well as residues V290, M277, Y317 and W319 localized in site-2 (figure 6*a,b*). Furthermore, the Y305A and M310A substitutions within binding sites-1 and -2 reduced binding to KRT10-TRD by 15% and 60%, respectively. Finally, the N321A substitution did not affect BR_187–385_-binding to KRT10-TRD, probably due to the fact that its side chain points towards the solvent instead of the predicted binding site. Importantly, the mutations affected neither the expression and solubility levels of the mutated proteins nor their retention volumes in SEC (data not shown). Furthermore, comparative circular dichroism (CD) spectra analysis of WT- and mutated BR_187–385_ proteins indicated that their overall secondary structure was not significantly affected by the introduced substitutions (see electronic supplementary material, figure S7). However, it cannot be excluded that the introduced substitutions may induce small conformational changes that were not detected by CD spectroscopy, but could affect binding to KRT10-TRD.

In conclusion, our results demonstrate that BR_187–385_ binds to KRT10-TRD using two contiguous paperclip-associated binding sites. Furthermore, binding of KRT10 to site-2 probably requires conformational adaptations by the front-loop.

### 3.5. Concluding remarks

In this study, the crystal structure of the KRT10-binding region domain (BR_187–385_) of PsrP was determined, revealing the compressed barrel fold as a member of the MSCRAMM family of adhesin proteins despite very low sequence identity. Our results suggest that electrostatic interactions may play an important role in initial complex formation. Indeed, the acidic helical rod domain of KRT10 could fit in the highly basic groove of BR_187–385_, created by an extended β-sheet on one side of the compressed barrel. Structural analysis and *in vitro* binding data also indicate the importance of the other face of the barrel that resembles a paperclip for binding to the tail-rod-2B region of KRT10. Future crystal structure determination of BR_187–385_ in complex with a KRT10-derived binding motif is required to elucidate the exact binding mechanisms, possibly confirming the importance of electrostatic interactions for initial complex formation.

## 4. Experimental procedures

### 4.1. Cloning

All protein constructs were cloned into the pET21d expression vector (Novagen) using the ligation-independent FastCloning method [30]. The coding sequence for the full-length basic region of PsrP (residues 2–395) was prepared from *S. pneumoniae* TIGR4 chromosomal DNA as described before [31] and used as template for PCR amplification to generate expression constructs comprising residues 187–385 of PsrP (BR_187–385_) with N-terminal poly-His (HHHHHH) and STII (SAWSHPQFEK) tags, respectively. Mutated expression constructs of BR_187–385_ were generated following previously described protocols [32].

The coding sequence for full-length KRT10 (clone ID HsCD00045373, Uniprot ID P13645) obtained from the DNASU repository [33] was PCR amplified to generate expression constructs of the head-rod-1A domain (KRT10-HRD: residues 1–179), tail-rod-2B domain (KRT10-TRD: residues 385–579), the rod domain (KRT10-ROD: residues 137–448, a M150L mutation was essential to prevent second translation initiation at ATG codon) and a full-length version (FL: 1–584) with N-terminal poly-His tag followed by a TEV cleavage site (HHHHHHENLYFQG; figure 5). All coding sequences of the protein-expression constructs were confirmed by DNA sequencing and are listed in the electronic supplementary material.

### 4.2. Expression, purification and optimization of protein constructs

Several poly-histidine-tagged constructs spanning different parts of the binding-region (BR) domain and with short length variations (of about six to eight residues) at both the N- and the C-termini were designed based on the overall domain organization of PsrP. Protein expression and solubility levels were checked using a small-scale expression test (http://tinyurl.com/EMBL-Heidelberg). The subdomain BR_187–385_ with higher crystallization probability was identified as the most promising construct using a limited proteolysis approach [34]. Protein expression was induced at OD 0.4–0.7 using 400 µM IPTG and performed overnight at 25°C. Expression of Seleno-methionine (Se-Met)-substituted protein was performed using Se-Met medium complete (Molecular Dimensions, UK) and Met-auxotroph *E. coli B834* cells (EMBL, Hamburg, Germany).

Poly-histidine-tagged BR_187–385_ was purified using immobilized metal affinity (IMAC) and cation exchange chromatography (CEC; HisTrap FF and HiTrap SPFF; GE Healthcare, Sweden). STII-tagged BR_187–385_ was purified using affinity chromatography on a Strep-Tactin superflow high-capacity column (IBA, Germany). Monomeric BR_187–385_ was eluted using SEC on Superdex 75 or 200 columns (GE Healthcare). The purity of poly-His- and STII-tagged BR_187–385_ constructs were assessed by SDS–PAGE to be at least 99% (see electronic supplementary material, figure S8*a*–*c*).

The soluble poly-His-tagged KRT10-TRD was purified using IMAC and anion exchange chromatography (AEC) on a 1 ml HiTrap Q HP (GE Healthcare). The poly-His-tagged KRT10-FL and KRT10-ROD were purified as inclusion bodies as described previously [35] and further purified in the presence of 6 M urea using AEC on a HiTrap Q HP 1 ml. KRT10-HRD was purified in the presence of 6 M urea using IMAC and CEC on a HiTrap Q SPFF 1 ml column (GE Healthcare). KRT10-FL, KRT10-ROD and KRT10-HRD were thereafter dialysed against urea-free buffer. The final purity of KRT10-TRD, KRT10-HRD, KRT10-ROD and KRT10-FL were estimated as 99%, 99%, 99% and more than 90% (see electronic supplementary material, figure S8*b*), respectively.

### 4.3. Crystallization of BR_187–385_

The BR_187–385_ monomer was concentrated to 20 mg ml^−1^ in 20 mM sodium citrate, 500 mM NaCl, 10% (v/v) glycerol, pH 5.5. Well-diffracting crystals of wild-type and Se-Met-BR_187–385_ were obtained in 0.2 M lithium sulfate, 0.1 M sodium acetate trihydrate pH 4.6, 25% PEG4000 (w/v) using the sitting drop vapour-diffusion method followed by micro-seeding. Crystals were cryo-protected by soaking in mother liquor supplemented with 25% glycerol and flash-frozen in liquid nitrogen.

### 4.4. Data collection and determination of the crystal structure of BR_187–385_

X-ray diffraction data from crystals of native and Se-Met-substituted BR_187–385_, both collected at beam line ID29 at the synchrotron radiation facility at the ESRF (Grenoble, France), were processed using the XDS program package [36] (table 1). The SAD dataset of a P4_3_2_1_2 Se-Met-substituted BR_187–385_ crystal diffracting to 2.25 Å was used to determine the crystal structure of BR_187–385_, based on the SAS protocol from Auto-Rickshaw [37]. Almost complete models for three BR_187–385_ molecules were obtained that were complemented through automatic rebuilding in Buccaneer [38]. Coot was used for all subsequent model building [39].

An MR search was performed using Phaser [40], and a single BR_187–385_ molecule was located in the asymmetric unit of the native crystal. Initial rigid body and restrained refinement rounds were performed in CCP4 Refmac [38] followed by model refinement using Phenix [41] with individual isotropic ADP factors and TLS refinement of the entire chain. A single Ramachandran plot outlier was found in the final model corresponding to residue T271, located in a β-turn motif with weak electron density. Finally, the side-chain atoms O^γ1^ and C^γ2^ of residue T378 were not built as a result of poor electron density.

The structural model was used to further refine the model corresponding to the anomalous dataset, using a simulated annealing protocol with subsequent LBFGS minimization with individual isotropic ADP factors, whole-chain TLS group refinement and NCS Cartesian restraints. At later stages, simulated annealing was omitted and NCS Cartesian restraints were altered to NCS torsion restraints. Crystal packing analysis revealed that the overall mobility of chain A (residues I204-S377) was relatively higher compared with the mobility of chains B and C (both comprising residues N203–S376), as reflected by higher overall B-factor values and lower map correlation coefficients (data not shown).

### 4.5. Structural analysis of BR_187–385_

The hydrophobicity of BR_187–385_ was assessed using the program package VASCo 1.0.2 [42]. PDB2PQR 1.7.1 [43] and APBS 1.3 in PyMOL [44] were used to calculate the electrostatic surface potentials. All figures were created using PyMOL version 1.3.0 [45]. Further programs used for structural analysis are listed in the electronic supplementary material.

### 4.6. Analytical ultracentrifugation analysis and sedimentation velocity experiments

Sedimentation velocity experiments were carried out on an analytical ultracentrifuge XLI (Beckman Coulter, Palo Alto, CA) with a rotor speed of 50 000 rpm, at 20°C, using a rotor Anti-50, and double-sector cells of optical path length 12 or 3 mm equipped with sapphire windows. Acquisitions were made using absorbance at 280 nm. Two samples of BR_187–385_ in 20 mM sodium citrate, 250 mM NaCl, 2.5% glycerol pH 5.5 were investigated. Solvent density of 1.017 g ml^−1^ and viscosity of 1.11 mPa s were measured at 20°C on density-meter DMA 5000 and viscosity-meter AMVn (Anton Paar), and the partial specific volume was estimated to 0.721 ml g^−1^ with the program SEDNTERP. The analysis was carried out in terms of distribution of sedimentation coefficients, *c*(*s*), and non-interacting species, with SEDFIT software, version 14.0c. The *c*(*s*) distributions showed a species at 1.85 S contributing to 97% of the total signal for two different sample concentrations of 45 and 22.5 µM. Their analysis in terms of one non-interacting species gave independent values for the molar mass of 23 and 20.5 kDa at the two concentrations, close to the theoretical value of 22.1 kDa. The theoretical sedimentation coefficients for 20 models from the generated EOM ensemble (see below) were calculated by the atomic-type/shell-model calculation in HYDROPRO [46] with a radius of the atomic elements of 2.9 Å.

### 4.7. Small angle X-ray scattering data processing and analysis

Synchrotron radiation X-ray scattering data were collected from five solute concentrations of BR_187–385_ in the range 1.1–8.7 mg ml^−1^ in 20 mM sodium citrate, 250 mM NaCl, 2.5% glycerol pH 5.5 were collected on the X33 camera of the EMBL on storage ring DORIS III (DESY, Hamburg, Germany) [47]. Data were collected using a photon counting Pilatus 1M detector at a sample–detector distance of 2.7 m and a wavelength of *λ* = 1.5 Å, the range of momentum transfer 0.01 < *s* < 0.6 Å^−1^ was covered (*s* = 4π sin*θ*/*λ*, where 2*θ* is the scattering angle). The forward scattering *I*(0), the radius of gyration *R*_g_ along with the pair distribution function of the particle *p*(*r*) and the maximum dimension *D*_max_ were computed by the automated SAXS data analysis pipeline [48]. The molecular mass (MM) of BR_187–385_ was evaluated by comparison of the forward scattering with that from a reference solution of bovine serum albumin (MM = 66 kDa). The excluded volume of the hydrated protein was computed with the program AUTOPOROD [49]. For globular proteins, the hydrated volumes in Å^3^ are about 1.6 times the MMs in Dalton. To assess the flexibility of BR_187–385_, the EOM [50] was used. See the electronic supplementary materials for details about data collection and analysis.

### 4.8. Pull-down assay

KRT10-FL was immobilized to 30 µl Ni–NTA magnetic agarose beads (Qiagen, Germany) in 10 mM potassium phosphate, pH 7.2, 250 mM NaCl, 0.05% Triton X-100 (magnetic PBS buffer). Beads not loaded with KRT10-FL protein were used as negative control. Beads were washed using 10 mM potassium phosphate, pH 7.8, 300 mM NaCl, 20 mM imidazole, 8% glycerol, 0.2% Triton X-100 (magnetic washing buffer) for 30 min. A volume of 100 µl of 25 µM STII-BR_187–385_ was incubated with the beads in 20 mM HEPES, 50 mM NaCl, 10% glycerol, pH 7.5 for 1 h. Beads were analysed using SDS–PAGE before incubation with BR_187–385_, after 10 min wash using magnetic PBS buffer, 1 min wash and three times of a 10 min wash using magnetic washing buffer.

### 4.9. ELISAs

ELISAs were performed using Nunc C96 MicroWell plates. PBS-T (PBS with 0.05% Tween-20) was used as washing buffer. Conjugates were detected using TMB liquid substrate system (Sigma Aldrich, USA). Coating levels of KRT10 constructs were detected using HRP-conjugated anti-His antibodies (anti-His AB-HRP, ab1187; Abcam, UK) and adjusted to the coating levels of KRT10-TRD incubated at a concentration of approximately 5 μg ml^−1^. 2% BSA (w/v) in PBS was used as blocking agent. STII-BR_187–385_ to KRT10 construct binding assays were performed in PBS with STII-BR_187–385_ concentrations ranging from 23 nM to 3 μM. For the second assay, WT-BR_187–385_ and mutated variants were used at a concentration of 1 μM for the BR_187–385_/KRT10-TRD interaction. Binding of STII-BR_187–385_ to KRT10 was detected using 250 ng μl^−1^ Strep-Tactin HRP conjugate in PBS-T (stock of IBA, Germany). Data were averaged and normalized as described in the electronic supplementary material.

## Supplementary Material

Supplemental material

## Data accessibility

PDB and structure factor files have been deposited under PDB ID codes 3ZGI and 3ZGH. Data that have not been deposited in a public repository will be made available upon request.
