# Correction to ‘The basic keratin 10-binding domain of the virulence-associated pneumococcal serine-rich protein PsrP adopts a novel MSCRAMM fold’

**DOI:** 10.1098/rsob.140172

**Published:** 2014-10-15

**Authors:** Tim Schulte, Jonas Löfling, Cecilia Mikaelsson, Alexey Kikhney, Karina Hentrich, Aurora Diamante, Christine Ebel, Staffan Normark, Dmitri Svergun, Birgitta Henriques-Normark, Adnane Achour

*Open Biol*. **4** (Published online 15 January 2014) (doi:10.1098/rsob.130090)

The residue numbers in the associated PDB files 3ZGH and 3ZGI were corrected during the PDB deposition process to have the same numbers as given in the associated Uniprot entry Q97P71. Unfortunately, we missed to update the residue numbers in the figures and text of the manuscript. The numbers in the manuscript should have an offset of −1. However, the first and last residue numbers given for the protein constructs BR_187–385_ and STII-BR_187–385_ remain valid.

