# Supplementary material for: The basic keratin 10-binding domain of the virulence-associated pneumococcal serine-rich protein PsrP adopts a novel MSCRAMM fold
Source: Open Biol. 2014 Jan 15;4(1):130090. doi: 10.1098/rsob.130090 (PMC3909270; doi:10.1098/rsob.130090)
Supplement: Supplemental material [file rsob130090supp1.pdf]

Supplemental Information

Supplemental figures:

Figure S1, related to figure 2)

SAXS-derived monomer models of $BR_{187-385}$ reveal highly flexible N- and C-terminal ends

Figure S2, related to figure 3)

Very low sequence identity between $BR_{187-385}$ and subdomains of Fap1, GspB and ClfB

Figure S3, related to figure 4)

$BR_{187-385}$ and ClfB have different KRT10-binding sites

Figure S4, related to figure 4)

The clip-associated loops have relatively high B-factor values, but a re-arrangement of their conformations requires breakage of hydrogen bond interactions between the front- and back-loop regions

Figure S5, related to figure 4) Basic groove of $BR_{187-385}$ could accommodate acidic helical rod of KRT10

Figure S6, related to figure 6)

Two binding sites within the KRT10 minimal binding domain of PsrP have the capacity to bind KRT10 head and tail domain-derived linear peptide motives

Figure S7, related to figure 6)

Circular dichroism spectra of wild-type and mutated STII- $BR_{187-385}$ proteins yield similar secondary structures

Figure S8, related to experimental procedures)

SDS-PAGE analysis of purified $BR_{187-385}$ and KRT10 proteins

Supplemental tables:

Table S1, related to figure 2) SAXS data collection and analysis parameters.

Table S2, related to figure 3) The top nine structural homologs to $BR_{187-385}$ are MSCRAMM and SRR proteins.

Supplemental movies, related to figure 3

Supplemental experimental procedures

Supplemental Figures

(a)

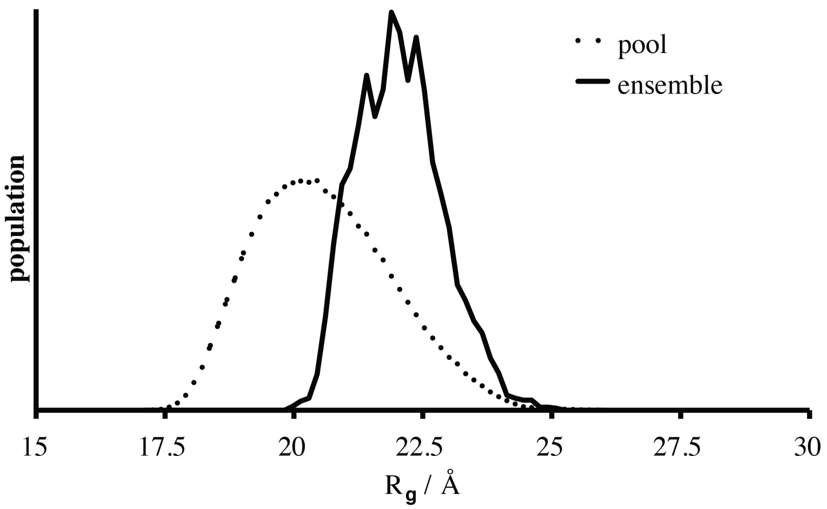

(b)

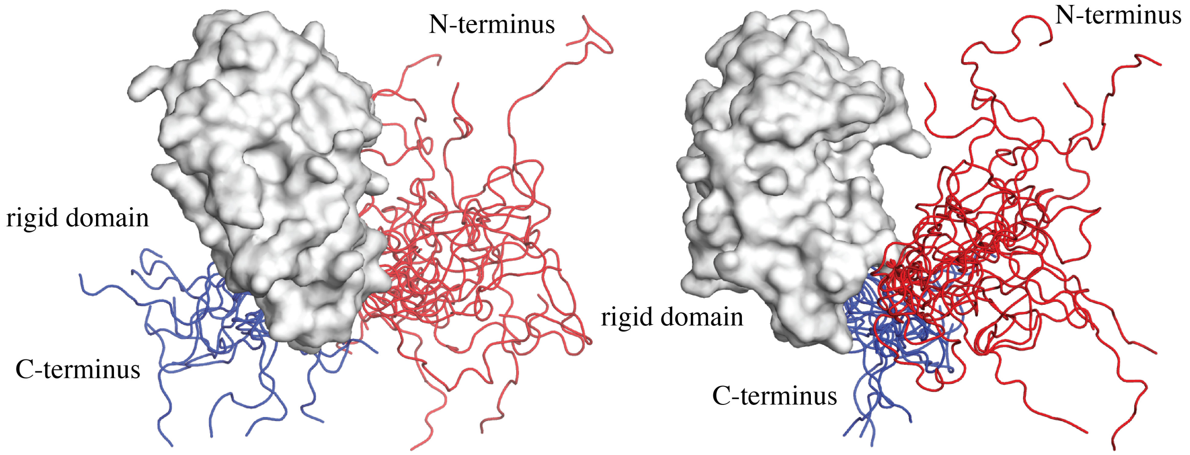

Figure S1, related to figure 2)

SAXS-derived monomer models of $BR_{187-385}$ reveal highly flexible N- and C-terminal ends

(a) The initial pool of 10.000 models with random configurations for the N and C-termini extending the rigid domain of $BR_{187-385}$ has a broad distribution of R_g values with a peak maximum at 20 Å (dotted line). The EOM ensemble is clearly biased towards extended structures with a mean R_g value of 22 Å (continuous line).

(b) Structural representations of the 18 models from the ensemble obtained by EOM analysis indicate high flexibility of the N- and C-terminal regions, which are drawn as thin lines in red and blue, respectively. The flexible terminal regions extend from the globular structure of $BR_{187-385}$, which is displayed as white molecular surface.

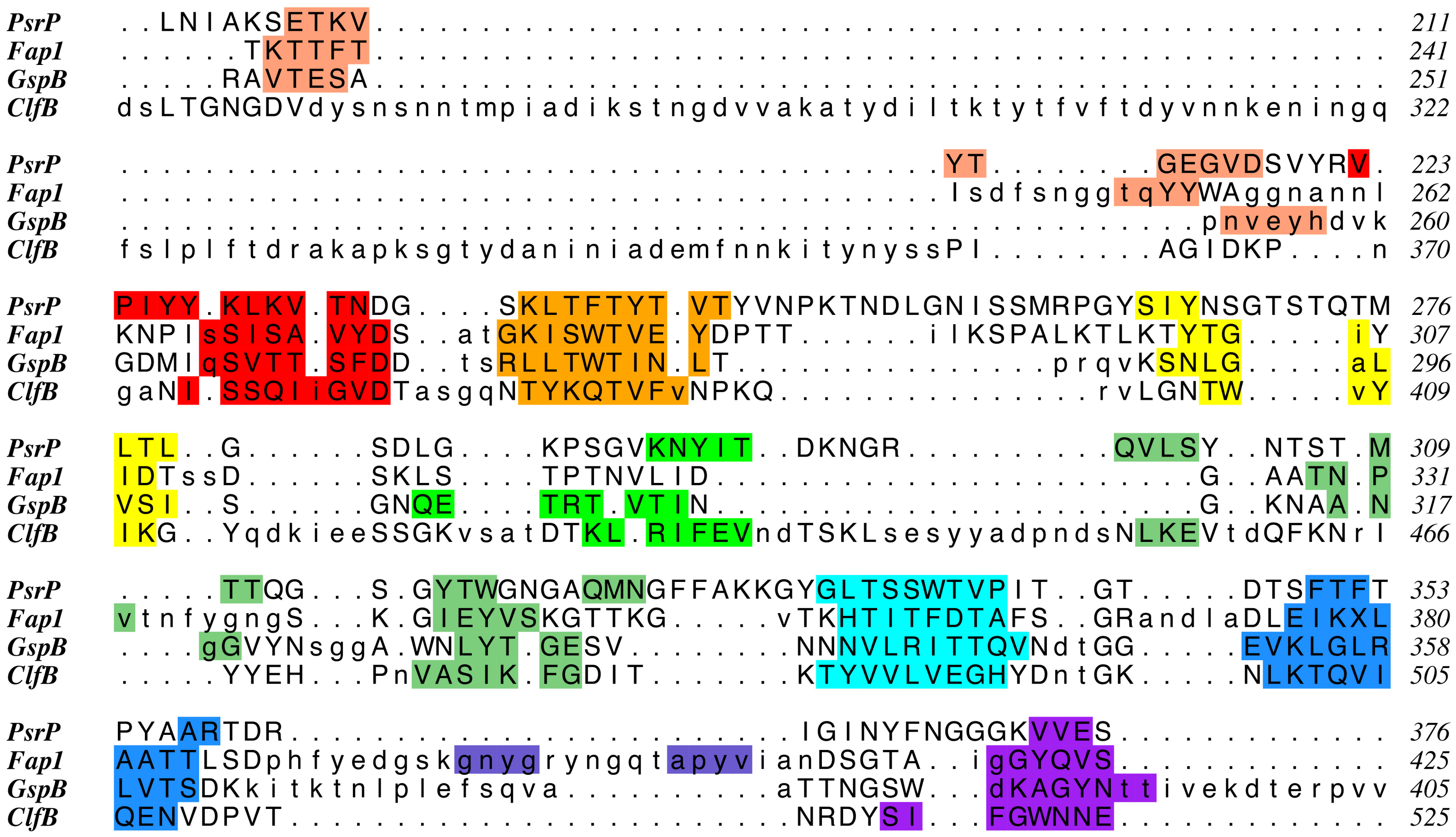

Figure S2, related to figure 3)

Very low sequence identity between $BR_{187-385}$ and subdomains of Fap1, GspB and ClfB

Sequence alignment was created using Dali [1]. Residues are color-coded according to figure 3. The amino acid sequence of $BR_{187-385}$ showed identities of only 5, 8 and 15% with the structurally aligned sequences of the N3 domain of ClfB (PDB code: 3AU0) and the two CnaA-subdomains of GspB (3QD1) and Fap1 (2X12), respectively.

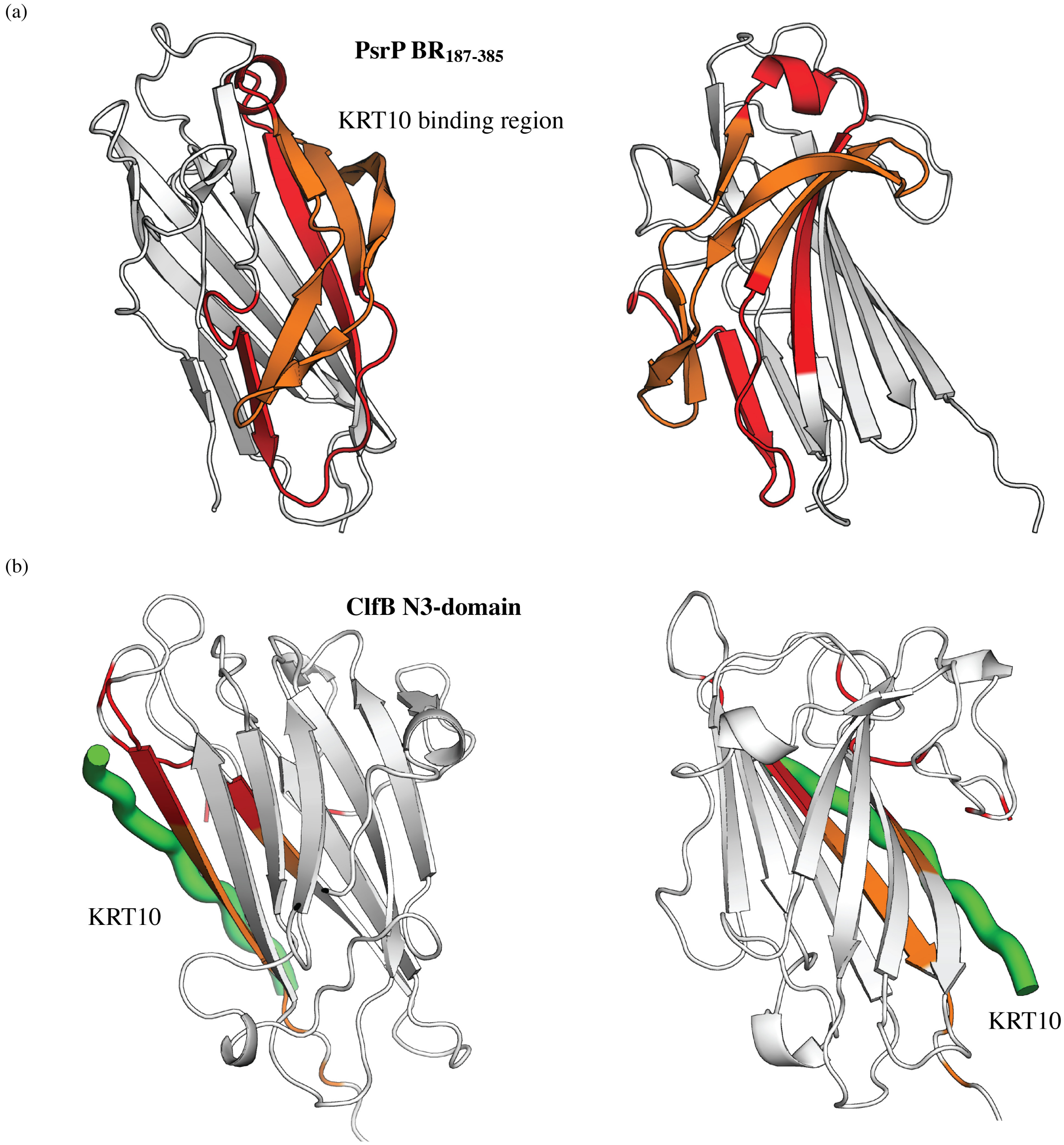
(a)
PsrP BR187-385
KRT10 binding region
(b)
ClfB N3-domain
KRT10
KRT10

Figure S3, related to figure 4)

$BR_{187-385}$ and ClfB have different KRT10-binding sites

The structures of $BR_{187-385}$ and ClfB, superimposed using Dali, are presented in similar orientations.

(a) The regions colored in orange and red correspond to the KRT10-binding region of $BR_{187-385}$. The figure is similar to figure 4a.

(b) Crystal structure of the N3-subdomain of ClfB in the apo-form. The position of the KRT10-derived peptide ligand (green) was obtained by superimposing the ClfB:KRT10 complex structure (PDB: 3ASW, [2]) onto the crystal structure of the Dali aligned ClfB apo-protein (3AU0). The KRT10 peptide ligand does not align with the protein region that would correspond to the KRT10-binding region of PsrP. Residues involved in hydrogen bond interactions with KRT10 in the KRT10/ClfB complex are colored orange. All remaining residues around KRT10 within a distance of 5 Å are colored red.

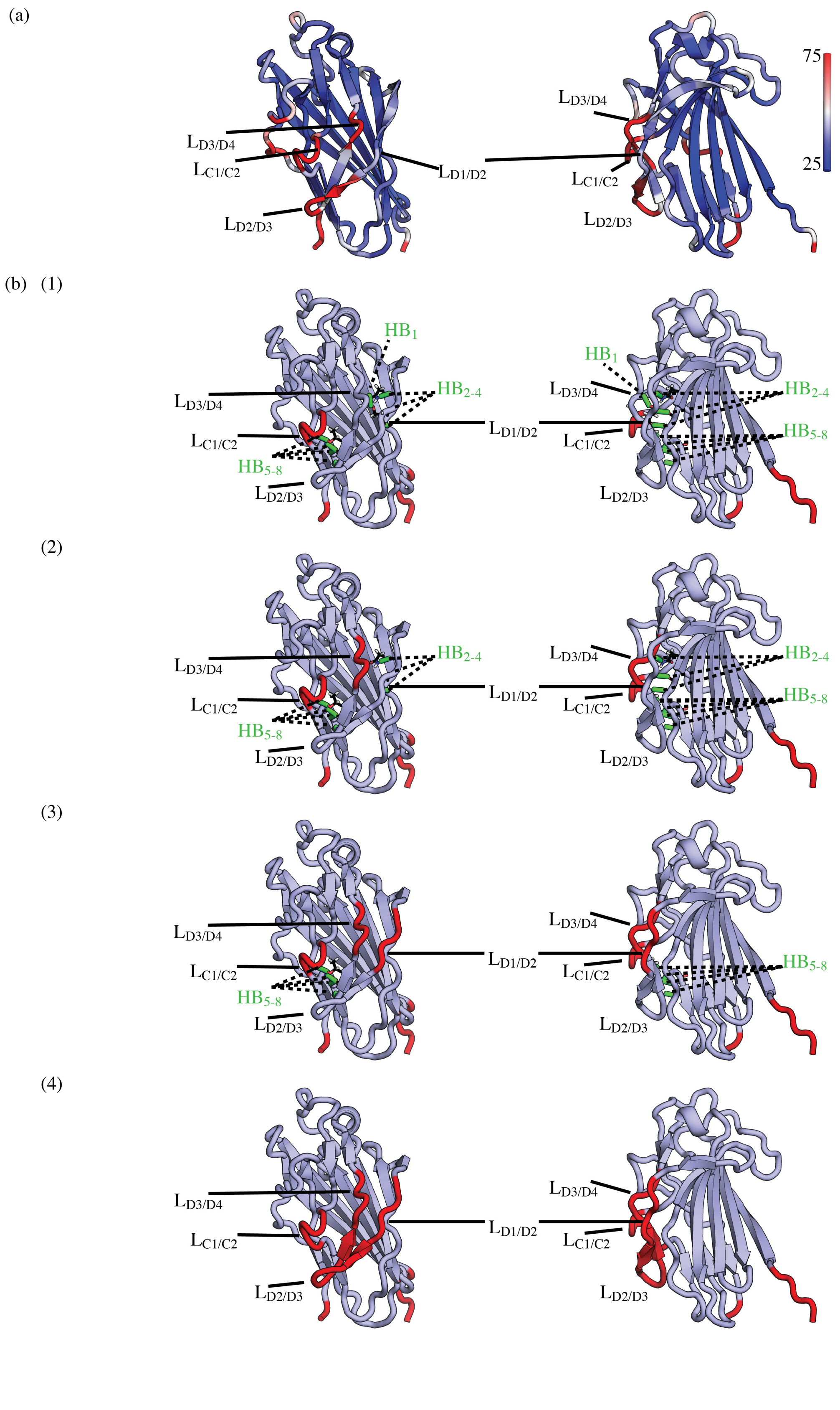

(a)
LD3/D4
LC1/C2
LD2/D3
LD1/D2
75
25
(b)
(1)
HB1
HB2-4
HB5-8
LD3/D4
LC1/C2
LD2/D3
LD1/D2
(2)
(3)
(4)

Figure S4, related to figure 4)

The clip-associated loops have relatively high B-factor values, but a re-arrangement of their conformations requires breakage of hydrogen bond interactions between the front- and back-loop regions

(a) Analysis of the B-factor distribution revealed highest flexibility for the clip-associated loops $L_{C1/C2}$, $L_{D3/D4}$ and $L_{D2/D3}$ localized at the tip of the front-loop. In contrast, the entire structure of $BR_{187\text{-}385}$ is much more rigid. The structure is colored according to the B-factor values (blue and red for low and high B-factors, respectively).

(b) Rigidity analysis of $BR_{187\text{-}385}$ identified the hydrogen bond (HB) interactions that couple the front-loop region to the large single rigid cluster of $BR_{187\text{-}385}$. These HB interactions are highlighted as green bars.

(1) The main rigid cluster of $BR_{187\text{-}385}$ is colored light blue. Residues that do not belong to the rigid cluster are colored red.

The HB interactions are listed according to the following: HB_1: Asn-321 CO - Ser-308 O^{γ}; HB_2: Asn-306 CO - Asn-292 $N^{\delta 2}$; HB_3: Ser-308 NH - Val-290 CO; HB_4: Ser-308 CO - Val-290 NH; HB_5: Thr-318 $O^{\gamma 1}$ - Gln-275 CO; HB_6: Met-277 NH - Tyr-317 CO; HB_7: Leu-278 NH - Tyr-317 CO; HB_8: Leu-278 CO - Tyr-317 NH.

(2) Removal of a single hydrogen bond interaction, HB_1, uncouples $L_{D3/D4}$ from the rigid cluster.

(3) Breakage of three hydrogen bond interactions ($HB_{2\text{-}4}$) uncouples $L_{D1/D2}$ from the rigid cluster.

(4) Breakage of four hydrogen bonds ($HB_{5\text{-}8}$) uncouples the entire front-loop.

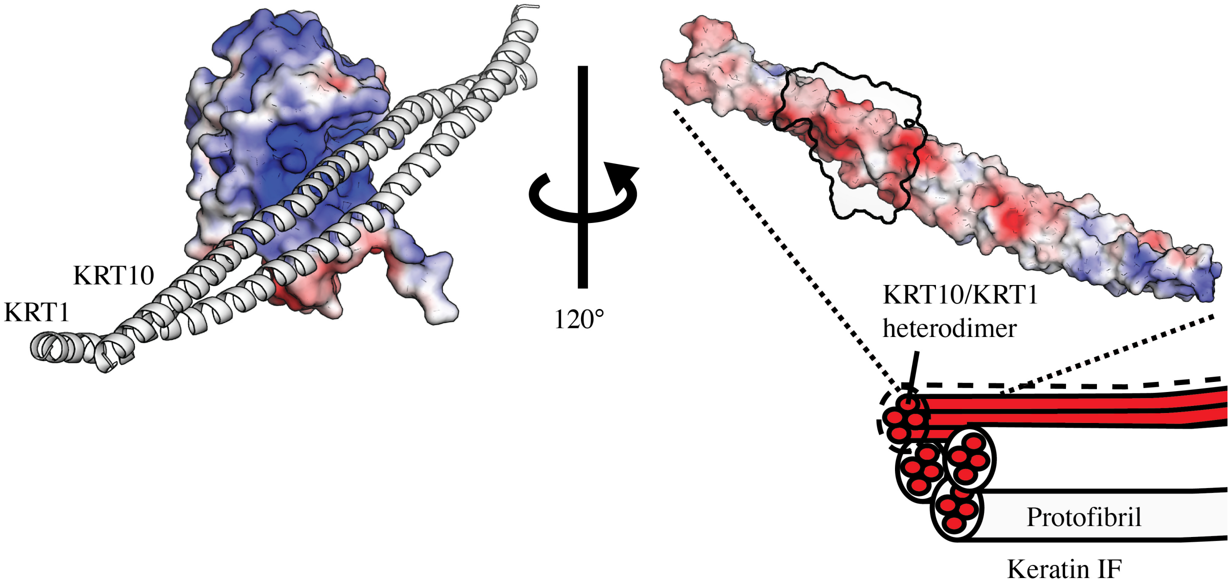

Figure S5, related to figure 4) Basic groove of $BR_{187\text{-}385}$ could accommodate acidic helical rod of KRT10

Docking prediction indicated a possible initial 'encounter' complex formation between the crystal structure of $BR_{187\text{-}385}$ and the homology model of an α-helical rod section within the KRT10/KRT1 intermediary filament. A schematic model illustrates the composition of the IF which is formed by four protofibrils, each composed of four KRT10/KRT1 heterodimers [3]. The electrostatic potentials of both ligands were plotted as in figure 4c. (Left) The α-helices of the KRT10/KRT1-heterodimer are shown in grey and the surface of $BR_{187\text{-}385}$ is colored according to its electrostatic potential. (Right) The electrostatic surface of the KRT10/KRT1 rod helices is represented. The surface of the $BR_{187\text{-}385}$ atoms that surround the helix with a distance cut-off at 8 Å is outlined in black.

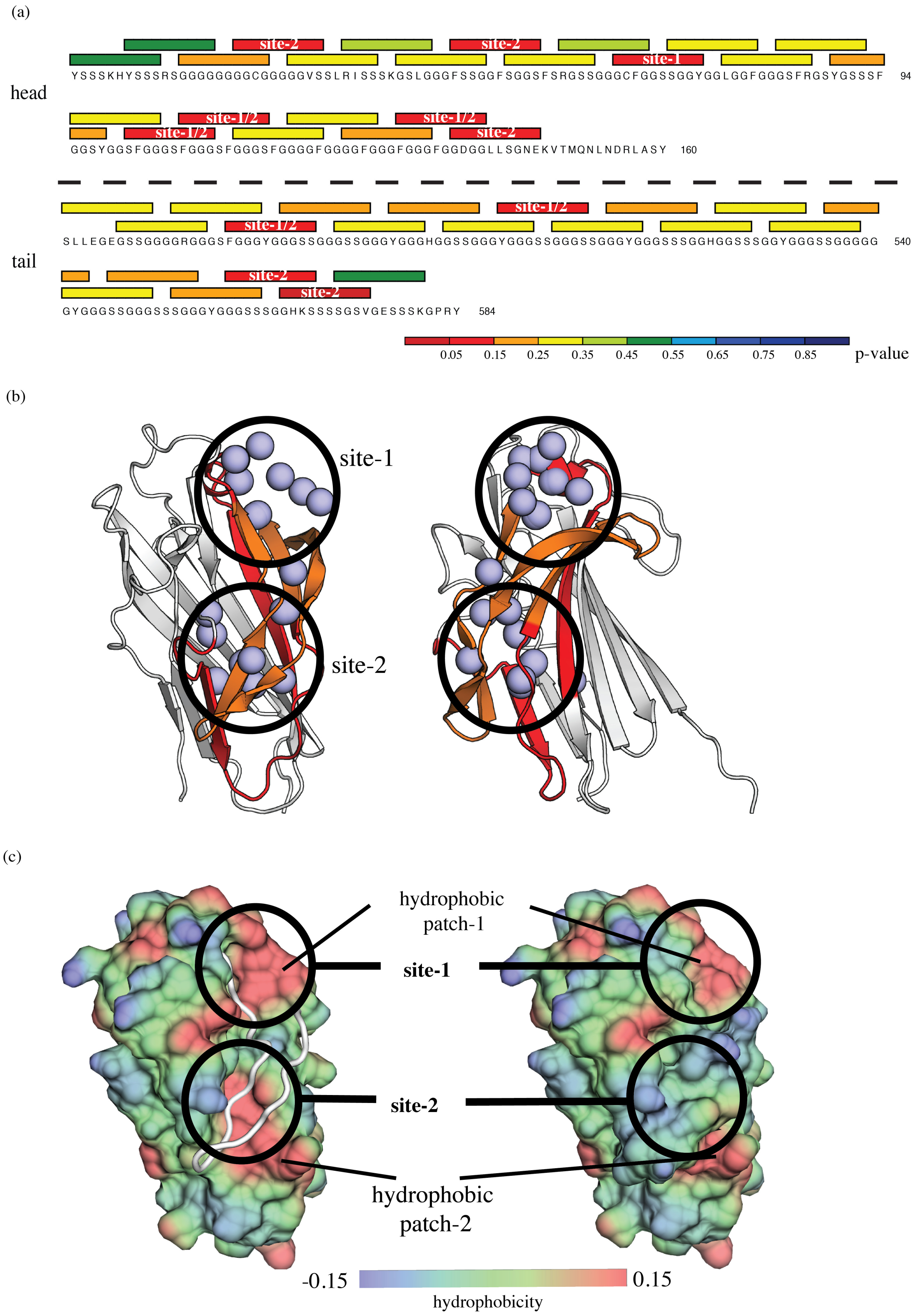
(a)
head
site-2
site-2
site-1
YSSSKHYSSSRSGGGGGGGGCGGGGGVSSLRISSSKGSLGGGFSSGGFSGGSFSRGSSGGGCFGGSSGGYGGLGGFGGGSFRGSYGSSSF
94
site-1/2
site-1/2
site-1/2
site-2
GGSYGGSFGGGSFGGGSFGGGSFGGGGFGGGGFGGGFGGGFGGDGGLLSGNEKVTMQNLNDRLASY
160
tail
site-1/2
site-1/2
SLLEGEGSSGGGGRGGGSFGGGYGGGSSGGGSSGGGYGGGHGGSSGGGYGGGSSGGGSSGGGYGGGSSSGGHGGSSSGGYGGGSSGGGGG
540
site-2
site-2
GYGGGSSGGGSSSGGGYGGGSSSGGHKSSSSGSVGESSSKGPRY
584
0.05
0.15
0.25
0.35
0.45
0.55
0.65
0.75
0.85
p-value
(b)
site-1
site-2
(c)
hydrophobic patch-1
site-1
site-2
hydrophobic patch-2
-0.15
0.15
hydrophobicity

Figure S6, related to figure 6)

Two binding sites within the KRT10 minimal binding domain of PsrP have the capacity to bind KRT10 head and tail domain-derived linear peptide motives

(a) Partially overlapping 10-residues long peptides (schematically represented by boxes above the amino acid sequence) were tested for binding to the KRT10 binding region of $BR_{187-385}$ using the PepSite server [4]. The boxes are color coded according to the peptide binding prediction values (lowest value = highest binding probability). A substantially higher amount of peptides derived from the head domain of KRT10 were predicted to bind to $BR_{187-385}$ compared to the tail domain. In general, peptides with high glycine/serine contents and with at least one hydrophobic residue were predicted to be strong binders.

(b) Two peptide-binding sites were predicted within the KRT10-binding region comprising residues 273-341 (orange and red). The space-filled atoms (light blue) represent the centers of mass of residues of KRT10-derived peptides that fitted into the two binding pockets with p-values < 0.15.

(c) The surface hydrophobicity distribution was calculated on $BR_{187-385}$ following removal of the front-loop of the clip (left panel). The hydrophobicity distribution of $BR_{187-385}$ is given for comparison (right panel). Hydrophobicity is shown using the same scale as in figure 4b.

(a)

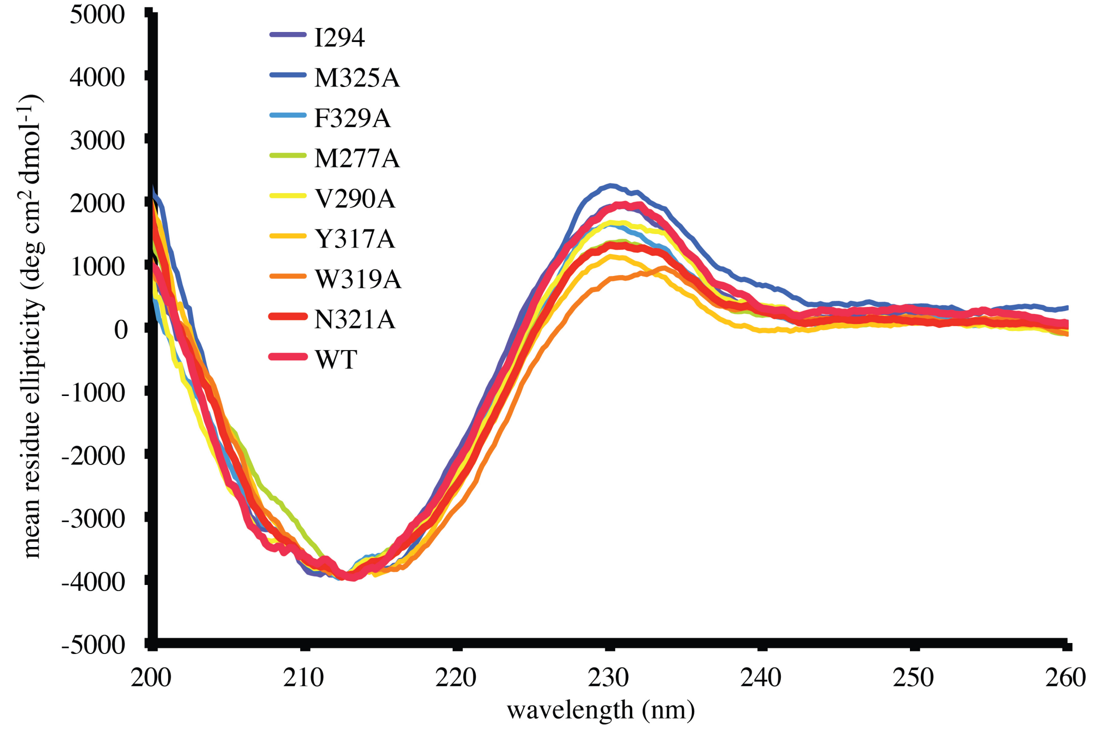

mean residue ellipticity (deg cm2 dmol-1)
5000
4000
3000
2000
1000
0
-1000
-2000
-3000
-4000
-5000
I294
M325A
F329A
M277A
V290A
Y317A
W319A
N321A
WT
200
210
220
230
240
250
260
wavelength (nm)

(b)

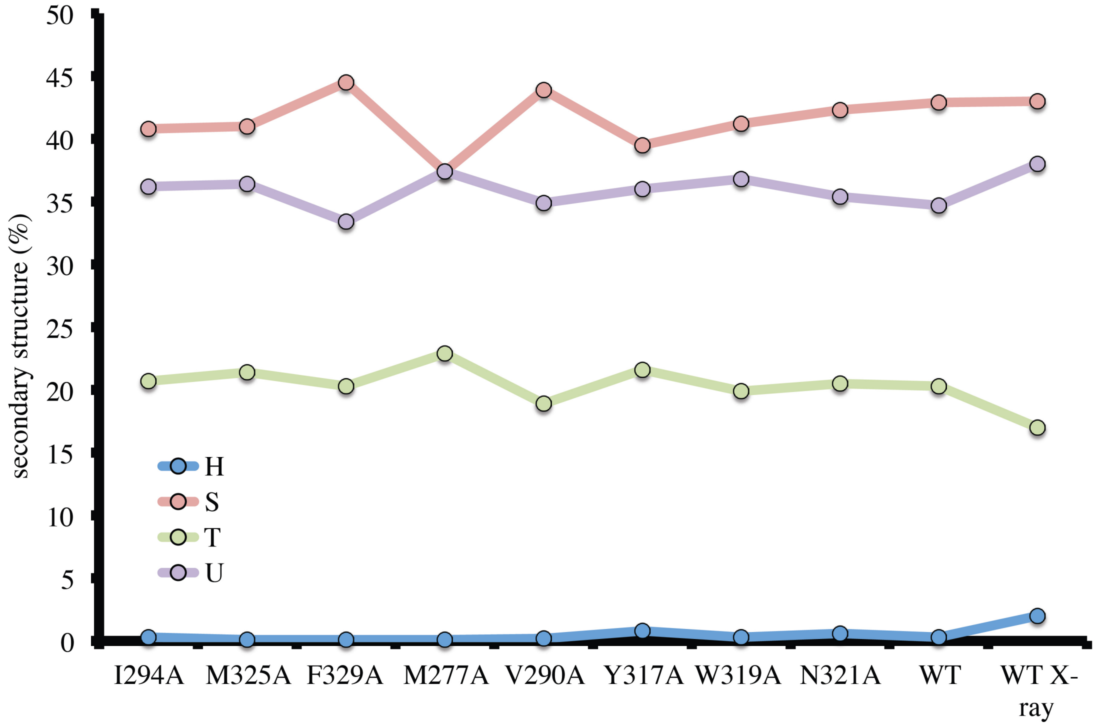

secondary structure (%)
50
45
40
35
30
25
20
15
10
5
0
H
S
T
U
I294A
M325A
F329A
M277A
V290A
Y317A
W319A
N321A
WT
WT X-ray

Figure S7, related to figure 6)

Circular dichroism spectra of wild-type and mutated STII-BR$_{187-385}$ proteins yield similar secondary structures

(a) CD spectra of WT and mutated STII-BR$_{187-385}$ proteins showed very similar CD spectral profiles with a negative peak extremum at 212 nm. The range of variability among all mutants is represented by spectral differences between wild-type STII-BR$_{187-385}$ and the N321A mutant, both binding to KRT10-TRD with the same intensity. The spectral profile of W319A showed the largest variation with a slight shift of the entire spectrum. However, the derived secondary structure element contributions were still in good agreement with the contributions derived from the crystal structure (see panel b).

(b) The average values of secondary structure contributions for all mutated and WT STII-BRLP protein samples are 42 ± 2% β-strands, 36 ± 1% disordered regions and 21 ± 1% turns. These values are in good agreement with the secondary structure contributions derived from the crystal structure with 43% β-strand, 38% unordered, 17% turns and 2% α-helices.

(a)

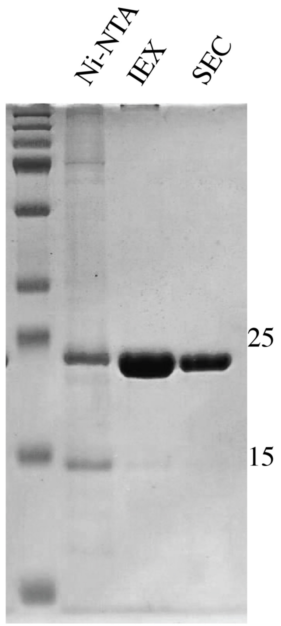
Ni-NTA
IEX
SEC
25
15

(b)

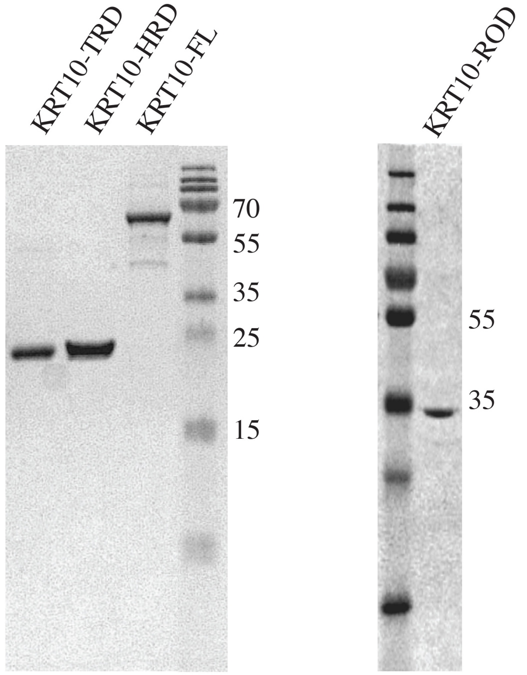
KRT10-TRD
KRT10-HRD
KRT10-FL
70
55
35
25
15
KRT10-ROD
55
35

(c)

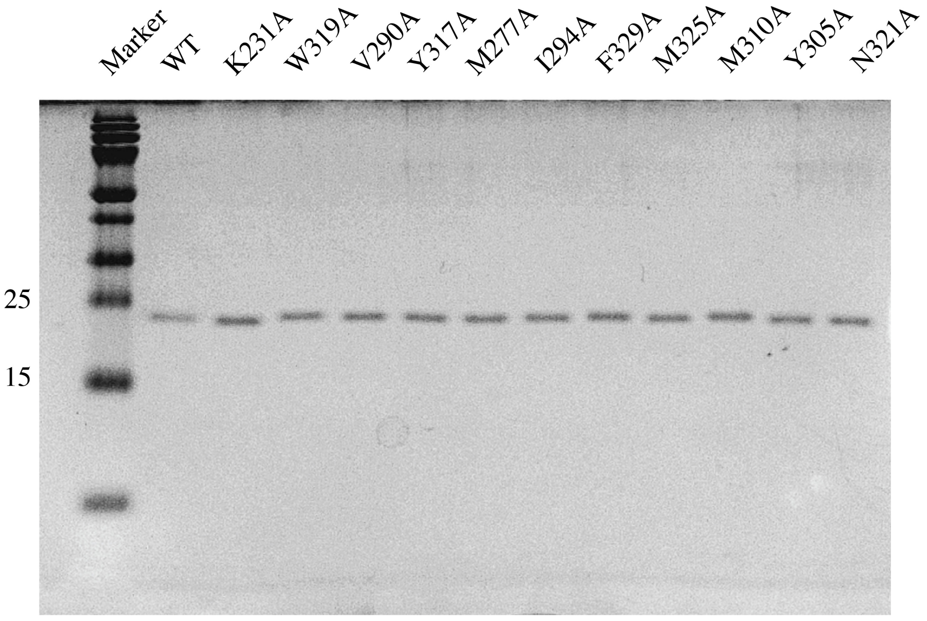
Marker
WT
K231A
W319A
V290A
Y317A
M277A
I294A
F329A
M325A
M310A
Y305A
N321A
25
15

Figure S8, related to experimental procedures)

SDS-PAGE analysis of purified $BR_{187-385}$ and KRT10 proteins

(a) SDS-PAGE analysis of poly-His tagged $BR_{187-385}$ (22.1 kDa) after Ni-NTA IMAC, ion-exchange chromatography (IEX) and size exclusion chromatography (SEC). The purity of poly-His-tagged $BR_{187-385}$ after SEC was estimated to 99%.

(b) SDS-PAGE analysis of poly-His tagged KRT10-TRD (19.6 kDa), KRT10-HRD (18.1 kDa), KRT10-ROD (37.6 kDa) and KRT10–FL (60.5 kDa) after final purification. The final purity of KRT10-TRD, KRT10-HRD, KRT10-ROD and KRT10-FL were estimated to 99%, 99%, 99% and more than 90%, respectively.

(c) SDS-PAGE analysis of WT STII-$BR_{187-385}$ (22.5 kDa) and all mutated variants following SEC purification. The purity of all the constructs was estimated to 99%.

All SDS-PAGE gels were stained using Coomassie Brilliant Blue R-250.

Table S1, related to figure 2) SAXS data collection and analysis parameters.

**data collection parameters**	
**instrument**	EMBL beamline X.33
**wavelength (Å)**	1.5
**s-range ($Å^{-1}$)**	0.01-0.6
**exposure time (s)**	15x8
**concentration range (mg/ml)**	1.1-8.7
**temperature (K)**	283
**structural parameters**	
**R_g (Å) (from Guinier)**	22.7 ± 1.2
**R_g (Å) (from p(r))**	22.5 ± 2.0
**D_{max} (Å)**	77.0 ± 8.0
**molecular mass determination**	
**Mw (kDa) from I(0)**	18 ± 2
**MM (kDa) from Porod volume**	23 ± 2
**calculated MM (kDa) from sequence**	22
**software employed**	
**primary data reduction and processing**	automated SAXS data analysis pipeline
**ab initio analysis**	DAMMIF
**flexibility analysis**	EOM

Table S2, related to figure 3) The top nine structural homologs to $BR_{187\text{-}385}$ are MSCRAMM and SRR proteins

protein (PDB code)	Z-score†	seq-id§ [%]	rmsd [Å]	residues aligned	comments [organism; protein family; fold of aligned domain; ligands]
**Fap1 2x12-A**	8.0	15	3.0	125	*Streptococcus parasanguinis*; SRRP; NRα domain with MSCRAMM fold; binds to the *in vitro* tooth model SHA [5]
**GspB 3qd1-X**	7.3	8	3.2	117	*Streptococcus gordonii*; SRRP; CnaA subdomain with MSCRAMM fold; binds to sialyl T-antigen via Siglec subdomain; [6]
**SpaA 3htl-X**	7.2	8	3.3	132	*Corynebacterium diphtheria*; pilus-subunit protein; M-domain with MSCRAMM fold [7]
**DT 1mdt-A**	7.2	6	4.7	119	*Corynebacterium diphtheria*; diphtheria toxin; R-domain with MSCRAMM fold [8,9]
**BP-2a-515 2xtl-B**	6.8	6	3.4	120	*Streptococcus agalactiae*; pilus subunit protein, D2 domain with MSCRAMM fold [10]
**Als9-2 2y7l-A**	6.7	17	3.4	110	*Candida albicans;* adhesin; N2 domain with MSCRAMM fold; binds to Fibrinogen-γ peptide [11]
**SdrG 1r17-A**	6.5	7	3.5	118	*Staphylococcus epidermis*; adhesin; N3 domain with MSCRAMM fold; binds to the Bβ chain of human fibrinogen [12]
**ClfB 3au0-A**	6.5	5	3.9	130	*Staphylococcus aureus;* adhesin; N3 domain with MSCRAMM fold; binds to keratin-10 [2][3][2](Ganesh et al., 2011)
**ClfA 1n67-A**	6.4	10	3.7	126	*Staphylococcus aureus*; adhesin, N3 domain with MSCRAMM fold; binds to fibrinogen [9]

† The Dali Z-score is a measure for structure similarity. Significant similarities correspond to similar folds and have a *Z*-score above 2; Strong matches of $BR_{187\text{-}385}$ would have a Z-score ≥ 16
§ The sequence identity is given for those residues that were structurally aligned.

Supplemental movies, related to figure 3)

The three movies illustrate the superimposition of $BR_{187\text{-}385}$ with the N3 domain of ClfB (movie 1, PDB code: 3AU0) and the two CnaA-subdomains of GspB (movie 2, 3QD1) and Fap1 (movie 3, 2X12). In each movie, $BR_{187\text{-}385}$ and the other proteins are colored black and red, respectively.

Supplemental experimental procedures

Translated coding sequences of His-BR$_{187-385}$, STII-BR$_{187-385}$, KRT10-FL, KRT10-ROD, KRT10-TRD and KRT10-HRD

> His-BR$_{187-385}$

MHHHHHHSGNTIVNGAPAINASLNIAKSETKVYTGEGVDSVYRVPIYYKLKVTNDGSKL
TFTYTVTYVNPKTNDLGNISSMRPGYSIYNSGTSTQTMLTLGSDLGKPSGVKNYITDKN
GRQVLSYNTSTMTTQGSGYTWGNGAQMNGFFAKKGYGLTSSWTVPITGTDTSFTFTPY
AARTDRIGINYFNGGGKVVESSTTSQSLSQ

> STII-BR$_{187-385}$

MSAWSHPQFEKSGNTIVNGAPAINASLNIAKSETKVYTGEGVDSVYRVPIYYKLKVTND
GSKLTFTYTVTYVNPKTNDLGNISSMRPGYSIYNSGTSTQTMLTLGSDLGKPSGVKNYIT
DKNGRQVLSYNTSTMTTQGSGYTWGNGAQMNGFFAKKGYGLTSSWTVPITGTDTSFTF
TPYAARTDRIGINYFNGGGKVVESSTTSQSLSQ

> KRT10-FL

MHHHHHHENLYFQGSVRYSSSKHYSSSRSGGGGGGGGCGGGGGVSSLRISSSKGSLGG
GFSSGGFSGGSFSRGSSGGGCFGGSSGGYGGLGGFGGGSFRGSYGSSSFGGSYGGSFGG
GSFGGGSFGGGSFGGGGFGGGGFGGGFGGGFGGDGGLLSGNEKVTMQNLNDRLASYL
DKVRALEESNYELEGKIKEWYEKHGNSHQGEPRDYSKYYKTIDDLKNQILNLTTDNANI
LLQIDNARLAADDFRLKYENEVALRQSVEADINGLRRVLDELTLTKADLEMQIESLTEEL

AYLKKNHEEEMKDLRNVSTGDVNVEMNAAPGVDLTQLLNNMRSQYEQLAEQNRKDA
EAWFNEKSKELTTEIDNNIEQISSYKSEITELRRNVQALEIELQSQLALKQSLEASLAETEG
RYCVQLSQIQAQISALEEQLQQIRAETECQNTEYQQLLDIKIRLENEIQTYRSLLEGEGSS
GGGGRGGGSFGGGYGGGSSGGGSSGGGYGGGHGGSSGGGYGGGSSGGGSSGGGYGG
GSSSGGHGGSSSGGYGGGSSGGGGGGYGGGSSGGGSSSGGGYGGGSSSGGHKSSSSGS
VGESSSKGPRY

> KRT10-ROD

MHHHHHHENLYFQGGDGGLLSGNEKVTLQNLNDRLASYLDKVRALEESNYELEGKIKE
WYEKHGNSHQGEPRDYSKYYKTIDDLKNQILNLTTDNANILLQIDNARLAADDFRLKYE
NEVALRQSVEADINGLRRVLDELTLTKADLEMQIESLTEELAYLKKNHEEEMKDLRNVS
TGDVNVEMNAAPGVDLTQLLNNMRSQYEQLAEQNRKDAEAWFNEKSKELTTEIDNNIE
QISSYKSEITELRRNVQALEIELQSQLALKQSLEASLAETEGRYCVQLSQIQAQISALEEQL
QQIRAETECQNTEYQQLLDIKIRLENEIQT

> KRT10-TRD

MHHHHHHENLYFQGLKQSLEASLAETEGRYCVQLSQIQAQISALEEQLQQIRAETECQN
TEYQQLLDIKIRLENEIQTYRSLLEGEGSSGGGGRGGGSFGGGYGGGSSGGGSSGGGYG
GGHGGSSGGGYGGGSSGGGSSGGGYGGGSSSGGHGGSSSGGYGGGSSGGGGGGYGGG
SSGGGSSSGGGYGGGSSSGGHKSSSSGSVGESSSK

> KRT10-HRD

MHHHHHHENLYFQGSVRYSSSKHYSSSRSGGGGGGGGCGGGGGVSSLRISSSKGSLGG
GFSSGGFSGGSFSRGSSGGGCFGGSSGGYGGLGGFGGGSFRGSYGSSSFGGSYGGSFGG
GSFGGGSFGGGSFGGGGFGGGGFGGGFGGGFGGDGGLLSGNEKVTMQNLNDRLASYL
DKVRALEESNYELEGKIK

Details about data processing and data analysis from SAXS experiments

To monitor radiation damage, eight successive fifteen-second exposures were compared and no significant changes were observed. The data were normalized to the intensity of the transmitted beam and radially averaged; the scattering of the buffer was subtracted and the difference curves were scaled for protein concentration. The low angle data collected from $BR_{187\text{-}385}$ were merged with the highest concentration high angle data to yield the final composite scattering curve.

The forward scattering I(0) and the radius of gyration R_g were evaluated using the Guinier approximation [13] assuming that at very small angles ($s < 1.3/R_g$) the intensity is represented as $I(s) = I(0)\exp(-(sR_g)2/3)$. During Ensemble Optimization Method (EOM) [14] analysis, a pool of 10000 models with a rigid domain comprising residues 207-376, as observed in the crystal structure of $BR_{187\text{-}385}$, and random configurations for the N- and C-termini were generated. A subset of the pool (18 models) was selected using a genetic algorithm such that the calculated averaged scattering of the selected models agreed with the experimental data. The R_g distributions of the selected ensembles were obtained by repeating the selection process multiple times.

Structural analysis of $BR_{187\text{-}385}$

Secondary structure analyses were performed using PDBsum [15] and 2Struc [16]. The rigidity were assessed using the KINARI webserver [17]. Interfaces of macromolecular assemblies in the crystal structures were investigated using PBDePISA [18]. The CASTp server [19] was used to identify possible binding pockets on the surface of $BR_{187\text{-}385}$. The KRT10-binding sites of $BR_{187\text{-}385}$ were predicted using the PepSite server version 1.0 [4]. A molecular model of the KRT10/KRT1 heterodimer (Uniprot IDs are P13645 and P04264, respectively), comprising residues 366-455 and 394-488, was created using the SWISS-MODEL homology server [20] and the crystal structure of the KRT14/KRT5 heterodimer [21] as a template. The geometry of the homology model was regularized using Phenix [22]. Complexes favored by van der Waals and electrostatic forces were obtained with the protein-protein docking server ClusPro 2.0 [23].

ELISA data analysis

Binding of STII-$BR_{187\text{-}385}$ to surface-coated KRT10-TRD, KRT10-HRD, KRT10-ROD and KRT10-FL

Data were averaged from eight ELISA assays. The BSA-background was subtracted and each value was normalized as follows:

$$x_{i,j}^{norm} = \frac{x_{i,j} - BSA_j}{FL, TRD_{max}}$$

with

$x_{i,j}$ = intensity of each measurement i for each STII- $BR_{187\text{-}385}$ concentration j

BSA_j= background binding of STII- $BR_{187\text{-}385}$ at concentration j to BSA

$FL,TRD_{max} = \frac{1}{2}[(FL_{j=max} - BSA_{j=FL,max}) + (TRD_{j=max} - BSA_{j=TRD,max})]$, maximum signal levels (at concentration j) measured for binding of STII- $BR_{187\text{-}385}$ to KRT10-FL and KRT10-TRD.

Binding curves were fitted using a single four-parameter logistic nonlinear regression model in Prism (GraphPad, USA).

Binding of STII- $BR_{187\text{-}385}$ and STII- $BR_{187\text{-}385}$ mutant proteins to KRT10-TRD

Data were averaged from three ELISA assays. In each ELISA, values were determined in quadruplicate, the BSA-background was substracted and normalized as follows:

$$x_{i,j}^{norm} = \frac{x_{i,j} - \overline{b_j}}{WT_{bg}^{norm}}$$

with

$x_{i,j}$ = intensity of each measurement i for each sample j

$\overline{b_j}$= averaged background binding of mutant j

$WT_{bg}^{norm} = \overline{(x_{i,j=WT} - \overline{b_{j=WT}})}$ = averaged, intensity-normalized binding of WT STII- $BR_{187\text{-}385}$

ANOVA analysis and a Tukey multiple comparison test at a significance level p > 0.05 were used to classify WT and mutated $BR_{187\text{-}385}$ into groups using Prism (GraphPad, USA).

Circular dichroism (CD) spectroscopy

Protein samples were diluted to give a final absorption corresponding to 0.05 in a 10 mm pathlength cuvette. Samples were analyzed in a 2 mm cuvette (Hellma, Germany) on a JASCO J-820 CD-Spectropolarimeter (Jasco, USA) at 25 °C. Three spectra were measured for each

sample (bandwidth 1.5 nm, step 0.2 nm, 2s response time), averaged and the spectrum of the dialysis buffer was subtracted. Data were converted to mean residue ellipticity according to formulas given in [24]. A 2nd polynomial Savitzky-Golay filter was applied over 15 data points using the software package Prism (GraphPad, USA). Spectra were scaled to each other at a wavelength of 212 nm (peak minimum). Secondary structure contributions were calculated using the CDSSTR algorithm with reference protein set 4 as implemented in the CDpro software package [25].

References

1 Holm, L., Kääriäinen, S., Rosenström, P. & Schenkel, A. 2008 Searching protein structure databases with DaliLite v.3. *Bioinformatics* **24**, 2780 –2781. (doi:10.1093/bioinformatics/btn507)

2 Ganesh, V. K. et al. 2011 Structural and biochemical characterization of Staphylococcus aureus clumping factor B/ligand interactions. *J. Biol. Chem.* **286**, 25963–25972. (doi:10.1074/jbc.M110.217414)

3 Parry, D. A. D. & Steinert, P. M. 1999 Intermediate Filaments: Molecular Architecture, Assembly, Dynamics and Polymorphism. *Quarterly Reviews of Biophysics* **32**, 99–187. (doi:null)

4 Petsalaki, E., Stark, A., García-Urdiales, E. & Russell, R. B. 2009 Accurate Prediction of Peptide Binding Sites on Protein Surfaces. *PLoS Comput Biol* **5**, e1000335. (doi:10.1371/journal.pcbi.1000335)

5 Ramboarina, S. et al. 2010 Structural insights into serine-rich fimbriae from gram-positive bacteria. *J. Biol. Chem* **285**, 32446–32457. (doi:10.1074/jbc.M110.128165)

6 Pyburn, T. M. et al. 2011 A Structural Model for Binding of the Serine-Rich Repeat Adhesin GspB to Host Carbohydrate Receptors. *PLoS Pathogens* **7**, e1002112. (doi:10.1371/journal.ppat.1002112)

7 Kang, H. J., Paterson, N. G., Gaspar, A. H., Ton-That, H. & Baker, E. N. 2009 The Corynebacterium diphtheriae shaft pilin SpaA is built of tandem Ig-like modules with stabilizing isopeptide and disulfide bonds. *Proc. Natl. Acad. Sci. U.S.A* **106**, 16967–16971. (doi:10.1073/pnas.0906826106)

8 Bennett, M. J. & Eisenberg, D. 1994 Refined structure of monomeric diphtheria toxin at 2.3 A resolution. *Protein Sci* **3**, 1464–1475. (doi:10.1002/pro.5560030912)

9 Deivanayagam, C. C. S., Wann, E. R., Chen, W., Carson, M., Rajashankar, K. R., Hook, M. & Narayana, S. V. L. 2002 A novel variant of the immunoglobulin fold in surface adhesins of Staphylococcus aureus: crystal structure of the fibrinogen-binding MSCRAMM, clumping factor A. *EMBO J* **21**, 6660–6672. (doi:10.1093/emboj/cdf619)

10 Nuccitelli, A. et al. 2011 From the Cover: Structure-based approach to rationally design a chimeric protein for an effective vaccine against Group B Streptococcus infections. *Proc. Natl. Acad. Sci. U.S.A* **108**, 10278–10283. (doi:10.1073/pnas.1106590108)

11 Salgado, P. S., Yan, R., Taylor, J. D., Burchell, L., Jones, R., Hoyer, L. L., Matthews, S. J., Simpson, P. J. & Cota, E. 2011 Structural basis for the broad specificity to host-cell ligands by the pathogenic fungus Candida albicans. *Proc. Natl. Acad. Sci. U.S.A.* **108**, 15775–15779. (doi:10.1073/pnas.1103496108)

12 Ponnuraj, K., Bowden, M. G., Davis, S., Gurusiddappa, S., Moore, D., Choe, D., Xu, Y., Hook, M. & Narayana, S. V. L. 2003 A 'dock, lock, and latch' structural model for a staphylococcal adhesin binding to fibrinogen. *Cell* **115**, 217–228.

13 Guinier, A. 1939 La Diffraction des rayons X aux très pétits angles: application à l'etude de phénomènes ultramicroscopiques ...

14 Bernadó, P., Mylonas, E., Petoukhov, M. V., Blackledge, M. & Svergun, D. I. 2007 Structural characterization of flexible proteins using small-angle X-ray scattering. *J. Am. Chem. Soc.* **129**, 5656–5664. (doi:10.1021/ja069124n)

15 Laskowski, R. A. 2009 PDBsum new things. *Nucleic Acids Res.* **37**, D355–359. (doi:10.1093/nar/gkn860)

16 Klose, D. P., Wallace, B. A. & Janes, R. W. 2010 2Struc: the secondary structure server. *Bioinformatics* **26**, 2624 –2625. (doi:10.1093/bioinformatics/btq480)

17 Fox, N., Jagodzinski, F., Li, Y. & Streinu, I. 2011 KINARI-Web: a server for protein rigidity analysis. *Nucleic Acids Research* **39**, W177–W183. (doi:10.1093/nar/gkr482)

18 Krissinel, E. & Henrick, K. 2007 Inference of macromolecular assemblies from crystalline state. *J. Mol. Biol* **372**, 774–797. (doi:10.1016/j.jmb.2007.05.022)

19 Dundas, J., Ouyang, Z., Tseng, J., Binkowski, A., Turpaz, Y. & Liang, J. 2006 CASTp: computed atlas of surface topography of proteins with structural and topographical mapping of functionally annotated residues. *Nucleic Acids Res.* **34**, W116–118. (doi:10.1093/nar/gkl282)

20 Arnold, K., Bordoli, L., Kopp, J. & Schwede, T. 2006 The SWISS-MODEL workspace: a web-based environment for protein structure homology modelling. *Bioinformatics* **22**, 195–201. (doi:10.1093/bioinformatics/bti770)

21 Lee, C.-H., Kim, M.-S., Chung, B. M., Leahy, D. J. & Coulombe, P. A. 2012 Structural basis for heteromeric assembly and perinuclear organization of keratin filaments. *Nat. Struct. Mol. Biol.* **19**, 707–+. (doi:10.1038/nsmb.2330)

22 Adams, P. D. et al. 2002 PHENIX: building new software for automated crystallographic structure determination. *Acta Crystallogr. D Biol. Crystallogr* **58**, 1948–1954.

23 Comeau, S. R., Gatchell, D. W., Vajda, S. & Camacho, C. J. 2004 ClusPro: an automated docking and discrimination method for the prediction of protein complexes. *Bioinformatics* **20**, 45–50.

24 Greenfield, N. J. 2007 Using circular dichroism spectra to estimate protein secondary structure. *Nat. Protocols* **1**, 2876–2890. (doi:10.1038/nprot.2006.202)

25 Sreerama, N. & Woody, R. W. 2000 Estimation of protein secondary structure from circular dichroism spectra: comparison of CONTIN, SELCON, and CDSSTR methods with an expanded reference set. *Anal. Biochem.* **287**, 252–260. (doi:10.1006/abio.2000.4880)
